# Genetic architecture of plant stress resistance: multi‐trait genome‐wide association mapping

**DOI:** 10.1111/nph.14220

**Published:** 2016-10-04

**Authors:** Manus P. M. Thoen, Nelson H. Davila Olivas, Karen J. Kloth, Silvia Coolen, Ping‐Ping Huang, Mark G. M. Aarts, Johanna A. Bac‐Molenaar, Jaap Bakker, Harro J. Bouwmeester, Colette Broekgaarden, Johan Bucher, Jacqueline Busscher‐Lange, Xi Cheng, Emilie F. Fradin, Maarten A. Jongsma, Magdalena M. Julkowska, Joost J. B. Keurentjes, Wilco Ligterink, Corné M. J. Pieterse, Carolien Ruyter‐Spira, Geert Smant, Christa Testerink, Björn Usadel, Joop J. A. van Loon, Johan A. van Pelt, Casper C. van Schaik, Saskia C. M. van Wees, Richard G. F. Visser, Roeland Voorrips, Ben Vosman, Dick Vreugdenhil, Sonja Warmerdam, Gerrie L. Wiegers, Joost van Heerwaarden, Willem Kruijer, Fred A. van Eeuwijk, Marcel Dicke

**Affiliations:** ^1^ Laboratory of EntomologyWageningen University and ResearchPO Box 166700 AAWageningenthe Netherlands; ^2^ Laboratory of Plant PhysiologyWageningen University and ResearchPO Box 166700 AAWageningenthe Netherlands; ^3^ Business Unit BioscienceWageningen Plant ResearchWageningen University and ResearchPO Box 166700 AAWageningenthe Netherlands; ^4^ Plant–Microbe InteractionsDepartment of BiologyUtrecht UniversityPO Box 800.563508 TBUtrechtthe Netherlands; ^5^ Laboratory of GeneticsWageningen University and ResearchPO Box 166700 AAWageningenthe Netherlands; ^6^ Laboratory of NematologyWageningen University and ResearchPO Box 82136700 ESWageningenthe Netherlands; ^7^ Wageningen University and Research Plant BreedingWageningen University and ResearchPO Box 3866700 AJWageningenthe Netherlands; ^8^ Section of Plant PhysiologySwammerdam Institute for Life SciencesUniversity of AmsterdamPO Box 942151090 GEAmsterdamthe Netherlands; ^9^ Section of Plant Cell BiologySwammerdam Institute for Life SciencesUniversity of AmsterdamPO Box 942151090 GEAmsterdamthe Netherlands; ^10^ Institute for Biology IRWTH Aachen UniversityWorringer Weg 352074AachenGermany; ^11^ BiometrisWageningen University and ResearchPO Box 166700 AAWageningenthe Netherlands

**Keywords:** abiotic stress, biotic stress, genetic architecture, genome‐wide association mapping, multiple stresses

## Abstract

- Plants are exposed to combinations of various biotic and abiotic stresses, but stress responses are usually investigated for single stresses only.
- Here, we investigated the genetic architecture underlying plant responses to 11 single stresses and several of their combinations by phenotyping 350 *Arabidopsis thaliana* accessions. A set of 214 000 single nucleotide polymorphisms (SNPs) was screened for marker‐trait associations in genome‐wide association (GWA) analyses using tailored multi‐trait mixed models.
- Stress responses that share phytohormonal signaling pathways also share genetic architecture underlying these responses. After removing the effects of general robustness, for the 30 most significant SNPs, average quantitative trait locus (QTL) effect sizes were larger for dual stresses than for single stresses.
- Plants appear to deploy broad‐spectrum defensive mechanisms influencing multiple traits in response to combined stresses. Association analyses identified QTLs with contrasting and with similar responses to biotic vs abiotic stresses, and below‐ground vs above‐ground stresses. Our approach allowed for an unprecedented comprehensive genetic analysis of how plants deal with a wide spectrum of stress conditions.

## Introduction

In nature, plants face variable environments that impose a wide range of biotic and abiotic stresses. These include, for example, below‐ground and above‐ground stresses, stresses imposed by unicellular and multicellular organisms, and short‐ and long‐lasting stresses. Under natural conditions, these stresses do not occur in isolation but are commonly present simultaneously (Rizhsky *et al*., 2004; Bergelson & Roux, 2010; Mittler & Blumwald, 2010; Vile *et al*., 2012; Prasch & Sonnewald, 2013; Rasmussen *et al*., 2013; Kissoudis *et al*., 2014; Rivero *et al*., 2014; Sewelam *et al*., 2014; Suzuki *et al*., 2014). Thus, plants are under strong selection to adapt to local conditions and have evolved sophisticated mechanisms to withstand multiple adverse environmental conditions (Howe & Jander, 2008; Bergelson & Roux, 2010; Pieterse *et al*., 2012; Stam *et al*., 2014; Brachi *et al*., 2015; Julkowska & Testerink, 2015; Kerwin *et al*., 2015). Yet, investigating this in a targeted experimental way is a major challenge owing to the complexity of multiple stress exposure. To gain an insight into the adaptation of plants to the wide variety of stress‐inducing conditions they face, genetic variation and mechanisms underlying stress resistance should be studied (Alonso‐Blanco *et al*., 2009; Brachi *et al*., 2015; Kerwin *et al*., 2015). The responses of plants to stresses have traditionally been investigated for individual stresses (Howe & Jander, 2008), but the research focus is currently shifting towards plant responses to combinations of stresses (Holopainen & Gershenzon, 2010; Pierik & Testerink, 2014; Stam *et al*., 2014; Suzuki *et al*., 2014; Kissoudis *et al*., 2015). The emerging picture is that responses to stress combinations cannot be predicted reliably from the responses to individual stresses (De Vos *et al*., 2006; Makumburage *et al*., 2013). For instance, the majority of transcriptional responses of *Arabidopsis* to combinations of two abiotic stresses could not be predicted from responses to the individual stresses (Rasmussen *et al*., 2013). Moreover, phenotype expression in response to two biotic stresses could not be predicted on the basis of existing information regarding interactions between underlying signaling pathways (De Vos *et al*., 2006). Phytohormones are major players in a signaling network, mediating responses to both biotic and abiotic stresses (Pieterse *et al*., 2009). For instance, chewing insect herbivores particularly elicit the jasmonic acid (JA), abscisic acid (ABA) and ethylene (ET) signaling pathways; phloem‐sucking insects and biotrophic microbial pathogens particularly elicit the salicylic acid (SA) pathway; and drought elicits the ABA pathway (Pieterse *et al*., 2009). The phytohormonal responses exhibit extensive crosstalk, resulting in specific changes in plant phenotype in response to individual stresses (De Vos *et al*., 2005; Pieterse *et al*., 2012).

In plant breeding, resistance and tolerance to multiple stresses are a common selection target (Braun *et al*., 1996). A well‐known strategy to achieve resistance and tolerance is by evaluation of candidate varieties in multi‐environment trials, that is, field trials at multiple locations during several years (van Eeuwijk *et al*., 2010; Malosetti *et al*., 2013). In such trials, multiple stresses can occur, but their occurrence and the intensity with which they occur are not guaranteed and, therefore, plant breeders developed the concept of managed stress trials in which specific and well‐defined stress conditions are imposed for a single stress or a small number of stresses (Cooper & Hammer, 1996; Cooper *et al*., 2014). Recently, the urge to manage environmental factors even more precisely has led to the development of phenotyping platforms, where, again, mainly single stresses are investigated (Fiorani & Schurr, 2013; Granier & Vile, 2014; Kloth *et al*., 2015).

Most studies, outside plant breeding, that have examined plant responses to multiple stresses included only one or a few genotypes (Holopainen & Gershenzon, 2010; Rasmussen *et al*., 2013; Pierik & Testerink, 2014; Stam *et al*., 2014; Suzuki *et al*., 2014; Kissoudis *et al*., 2015). To obtain a further understanding of the genetic architecture of complex traits such as plant adaptation to a diversity of stresses, extensive study of the natural genetic variation within a species is instrumental. Genome‐wide association (GWA) analysis is an important tool for this, requiring a large number of well‐genotyped plant accessions. Yet, although the interest in natural variation and GWA mapping is rapidly increasing (Wijnen & Keurentjes, 2014; Ogura & Busch, 2015), a large‐scale evaluation of natural genetic variation in resistance of plants to the full diversity of stresses to which they are exposed, including pathogens, herbivores and abiotic stresses and their interactions, has not been done to date. To elucidate the genetic architecture of plant stress resistance, an integrated approach is needed that models the genetics of responses to a range of single and combined stresses, including the interaction between those responses. Here, we have adopted a comprehensive and integrated approach to investigate the genetics underlying plant responses to 15 carefully standardized single stresses or stress combinations (Table 1), making use of a global population of 350 *Arabidopsis* accessions that have been genotyped for 214 000 single nucleotide polymorphisms (SNPs) (Baxter *et al*., 2010; Li *et al*., 2010). The standardization of these 15 stress conditions is an important element of the study, because it allows for phenotyping of well‐defined stress responses. We developed a tailored multi‐trait GWA analysis that allowed the identification of candidate genes associated with plant responses to multiple stresses that were validated by gene expression and mutant analyses.

**Table 1:**
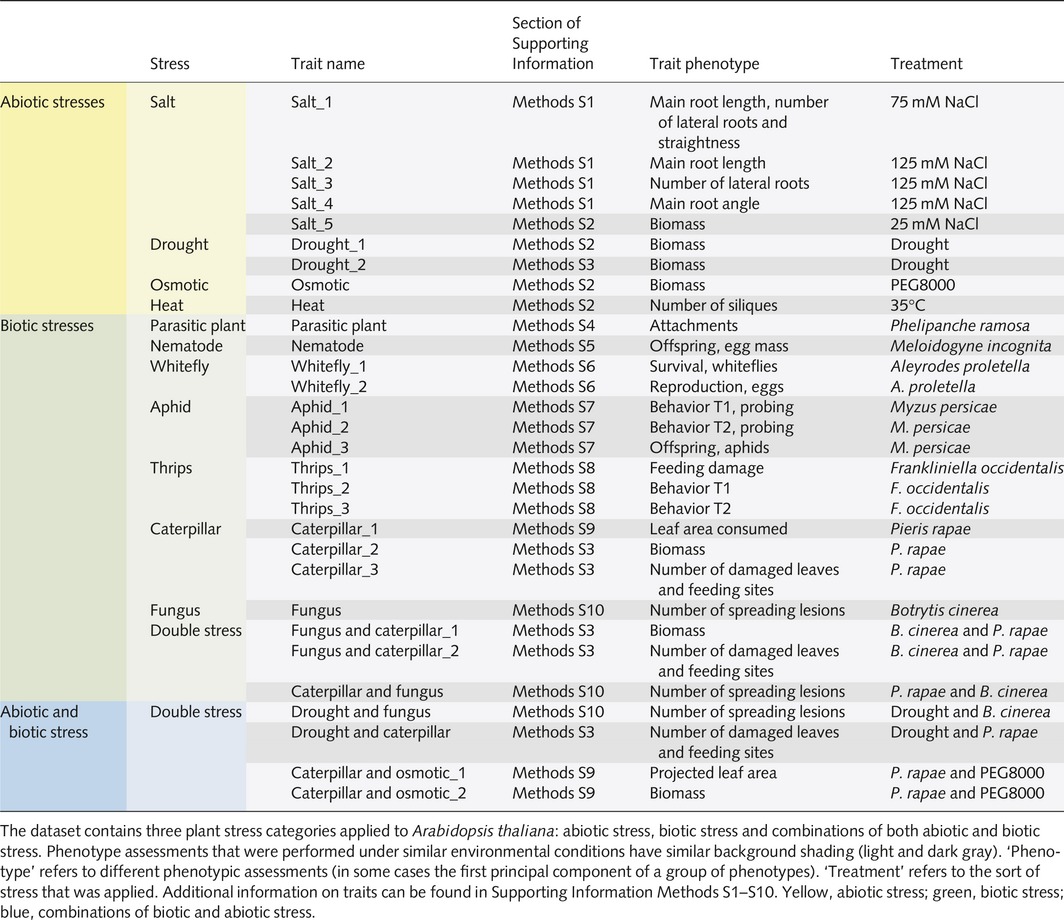
Phenotypes assessed

## Materials and Methods

### *Arabidopsis thaliana* population

In this study we included 350 *Arabidopsis thaliana* (L.) Heynh. accessions from the HapMap population (http://bergelson.uchicago.edu/wp-content/uploads/2015/04/Justins-360-lines.xls). The HapMap population has been genotyped for 250 000 bi‐allelic SNPs (Baxter *et al*., 2010; Platt *et al*., 2010; Chao *et al*., 2012) and after quality control and imputation this SNP set was reduced to a set of 214 051 SNPs.

### Definition of the target traits

For every experiment, the target traits were derived from the individual plant data using the following strategy. First, when residuals deviated from normality, a logarithmic, arcsine or square root transformation was applied to the original observations. Second, genotypic (accession) means for each treatment were calculated using a mixed model to account for design effects. Different mixed models were used in the experiments, reflecting the different designs. In all cases, accession effects were modeled as fixed, and the accession means were the best linear unbiased estimator (BLUE) of these effects. Third, for traits measured in treatment and control conditions, differences or residuals (when regressing treatment on control values) were defined, in order to obtain a measure of stress tolerance that was corrected for the expression of the same trait under control conditions. Finally, within each experiment, the traits were replaced by the first principal component if the latter explained more than half of the variation in all traits in this experiment; in all other cases, the original traits were retained. An overview of final traits and their corresponding sections in the Supporting Information Methods can be found in Table 1. In case of replacement by the first principal component, original traits and the variance explained by the first principal component are listed (Supporting Information Methods Tables M1–M5 in Methods S1–S3, S7 & S9). In total, phenotypic data for 73 individual traits were obtained by 10 different research groups. All calculations were performed in R, unless stated otherwise. Mixed‐model analysis was performed with the R package ASReml (Butler *et al*., 2009). In all equations, the term *E* denotes residual error. All other terms represent fixed effects unless stated otherwise. A colon (:) is used to define interactions between terms.

### Statistics

#### Genetic correlation networks and heritability

Pairwise marker‐based genetic correlations between traits, genomic correlations, were estimated using a multi‐trait mixed model (MTMM) (Korte *et al*., 2012). Residuals were assumed to be uncorrelated for traits that were measured on different plants. For some pairs of traits, the likelihood was monotone, which can also occur in single‐trait mixed models (Kruijer *et al*., 2015). In this case, the genetic correlation was estimated by the (Pearson) correlation between the univariate G‐BLUPs (De los Campos *et al*., 2013) estimated for these traits. A network between predefined groups of traits was constructed by connecting groups whose average genetic correlation across pairs of traits was > 0.2.

Narrow sense heritability (Table S1) was estimated using the mixed model *Y*_*i*_ = *μ* + *A*_*i*_ + *E*_*i*_ where *Y*_*i*_ represents the phenotypic means of accessions (*i* = 1, …, 350), and *A*_*i*_ and *E*_*i*_ are random genetic and residual effects. The vector of additive genetic effects follows a multivariate normal distribution with covariance $\sigma_{A}^{2}$ *K*,* K* being a marker‐based relatedness matrix. The residual errors are independent, with variance $\sigma_{E}^{2}$. We obtained restricted maximum likelihood (REML) estimates of $\sigma_{A}^{2}$ and $\sigma_{E}^{2}$, and estimated heritability as $h^{2}=\sigma_{A}^{2}/(\sigma_{A}^{2}+\sigma_{E}^{2}$). This is an estimate of narrow‐sense heritability, as the model for the genetic effects only captures additive effects, and $\sigma_{E}^{2}$ is the sum of environmental and nonadditive genetic effects (see e.g. Kruijer *et al*., 2015).

#### Multi‐trait mixed models

Following Zhou & Stephens (2014), we assume the MTMM, *Y *= *XB* + *G *+ *E*, with *Y* being the genotypes by traits (*n *×* p*) matrix of phenotypic observations. The terms *XB*,* G* and *E* stand for, respectively, the fixed effects (including trait‐specific intercepts and SNP effects) and the random genetic and environmental effects. *G* follows a zero mean matrix‐variate normal distribution with row‐covariance (marker‐based kinship) matrix *K* and column (trait) covariance matrix *V*_g_. *V*_g_ is a *p* × *p* matrix modeling the genetic correlations between traits. This is equivalent to g = *vec*(*G*) (the vector containing the columns of *G*) being multivariate normal with a covariance matrix defined by the Kronecker product *V*_g_ ⊗ *K* (Zhou & Stephens, 2014). Similarly, *vec*(*E*) follows a zero mean normal distribution with covariance *V*_e_ ⊗ *I*_n_, where *V*_e_ accounts for the nongenetic correlations between traits.

#### Factor‐analytic models

As *V*_g_ and *V*_e_ contain a total of *p*(*p *+* *1) parameters, the MTMM becomes difficult to fit for > 10 traits (Zhou & Stephens, 2014). For *V*_g_ we therefore assumed a factor analytic model, which is well known in the context of quantitative trait locus (QTL) mapping for experimental populations with limited numbers of markers (Boer *et al*., 2007), but has not been used in the context of multivariate GWA studies (GWAS). As almost all traits were derived from measurements on different plants, a diagonal model $V_{e}=diag\left(\sigma_{e,1}^{2},\ldots ,\;\sigma_{e,p}^{2}\right)$ was chosen for the environmental covariances. For *V*_g_, a second‐order factor analytic structure was chosen $V_{g}=\sigma_{g}^{2}\left(\lambda \lambda^{t}+diag\left(\tau_{1}^{2},\ldots ,\;\tau_{p}^{2}\right)\right)$, where $\sigma_{g}^{2}$ represents a scale parameter, the magnitude of genetic effects, the *p × *2 matrix λ contains the trait‐specific scores belonging to the factor analytic part of the model that provides a rank two variance‐covariance structure between traits, and $diag(\tau_{1}^{2},\ldots ,\tau_{p}^{2})$ provides trait‐specific residual genetic variances (Piepho, 1997; Meyer, 2009). The model was fitted with the R package ASRreml (Butler *et al*., 2009).

#### Compressed kinship

Factor analytic models have been successfully applied to experimental populations with a simple genetic relatedness structure (Boer *et al*., 2007; Malosetti *et al*., 2008; Alimi *et al*., 2013), but currently available software could not perform REML estimation for the HapMap population. The kinship matrix was therefore replaced with a compressed kinship matrix (Bradbury *et al*., 2007; Zhang *et al*., 2010), modeling the genetic relatedness between a number of internally homogeneous groups. Assuming there are *m* such groups, containing *n*_1_, …, *n*_*m*_ accessions each, the original kinship matrix *K* is replaced by *ZK*_C_ *Z* ^*t*^, where *K*_C_ is the kinship matrix for the groups, and *Z* is the *n × m* incidence matrix assigning each of the *n* accessions to one of the *m* groups. The groups were created by a procedure that restricted the marker data to be linear combinations of environmental covariates representing the conditions at the place of origin of the accessions, as explained later.

Compressed kinship was calculated as the average kinship within genetic groups. Genotypes were assigned to *k* genetic groups by performing Ward clustering based on the squared Euclidean distance along the first *k* − 1 principal components calculated from a matrix of standardized SNP scores, followed by cutting the resulting dendrogram into *k* distinct clusters (van Heerwaarden *et al*., 2012, 2013; Odong *et al*., 2013).

The use of a compressed kinship matrix requires a choice of the degree of compression, as determined by the number of genetic groups over which the individual kinship is averaged. This choice needs to balance the gain in computational efficiency with model fit (Zhang *et al*., 2010) and the ability of the compressed matrix to capture the correlation between genetic dissimilarity and phenotypic differences, which is ultimately the reason for including a kinship matrix in the association model. There are currently no standard methods to determine the optimum degree of compression, at least not when used in a multi‐trait setting. We determined the appropriate degree of compression for each association model based on the model likelihood, convergence and correspondence between kinship and phenotypic and geographical similarity. The latter was quantified as the Frobenius norm of the difference between the complement of the compressed kinship matrix, expanded to a block matrix of full rank, and the Euclidean distance matrix of phenotypic traits or geographic coordinates. We considered a range of four to 100 groups. Correspondence with phenotypic and geographical dissimilarity increased steeply from four to *c*. 35 groups, after which correspondence with geographic distance increased more slowly and the correspondence with phenotypic distance showing a local decrease until 58 groups. Model likelihood was relatively stable above four groups, but convergence was erratic depending on the modeled contrasts. For each model the number of groups was therefore chosen to be the minimum number of groups needed to achieve a degree of correspondence approximating that found at 35 groups, under condition of model convergence.

#### Multi‐trait GWAS

Traits (columns of *Y*) were standardized. Along the genome, MTMMs of the type *Y *= *XB* + *G* + *E* were fitted with initially for each marker trait‐specific QTL effects *β*_1_, …, *β*_*p*_ (contained in *B*). To identify general QTLs with trait‐specific effects, for individual markers, the null hypothesis *β*_1_ **= *β*_2_ **= … *β*_*p*_ **= 0 was tested by a Wald test against the alternative hypothesis that at least one of the trait‐specific effects was nonzero (Zhou & Stephens, 2014). To identify consistent QTLs, the null hypothesis *β*_1_ **= *β*_2_ **= … *β*_*p*_ = *β* ≠ 0 was tested. To identify potentially adaptive QTLs, contrasts defined on the trait‐specific QTL effects were tested. For example, suppose the first *p*1 of the full set of *p* traits represents responses measured under abiotic stresses, while the second *p*2 traits represent responses under biotic stresses. A contrast can now be defined to test the hypothesis of whether the QTL effect for abiotic stresses differs from that for biotic stresses: *β*_1_ **= *β*_2_ **= … *β*_*p*1_ **= *α*_abiotic_; *β*_*p*1+1_ = *β*_*p*1+2_ **= … *β*_*p*_ **= *α*_biotic_ and *H*_0_: *α*_abiotic_ = *α*_abiotic_ vs *H*_a_: *α*_abiotic_ ≠ *α*_biotic._ For the Wald test for the hypothesis *β*_1_ **= …* *= *β*_*p*_ we first fit the MTMM *Y *= *XB* + *G *+ *E* with *XB* only containing trait‐specific means *μ*_1_, …, *μ*_*p*_, and then we test hypotheses on the marker effects. The contrast is defined through a partitioning of the traits in two groups (e.g. resistance against biotic or abiotic stress). Using the R package ASReml (Butler *et al*., 2009) we perform Wald tests for the following hypotheses:

1. *H*_0_: *β* = 0, in the constrained model *β*_1_ **= …* *= *β*_*p*_ **= *β*.
2. *H*_0_: *α*_1_ **= *α*_2_, in the constrained model where *α*_1_ is the effect on all traits in the first group, and *α*_2_ is the effect on traits in the second group.

#### Simulations to compare power for full MTMM, contrast MTMM and univariate analysis

We further compared the different Wald tests using simulations, described in more detail in Methods S12. Specifically, we compared the performance of the general MTMM (i.e. testing the hypothesis *β*_1_ = *β*_2_ = … *β*_*p*_ = 0) with the MTMM used for the contrasts (i.e. *H*_0_: *α*_group1_ = *α*_group2_, where, within two predefined groups of traits, all SNP effects equal *α*_group1_ and *α*_group2_, respectively). We simulated phenotypic data for given genotypic data, either assuming the SNP effects were positive (but not equal) within one group of traits and negative for the other (scenario A), or choosing the sign of each SNP effect randomly (scenario B). The simulation results as presented in Fig. S11 (see later) clearly indicate that the Wald test for the contrast has superior power under scenario A, while the general MTMM performs best under scenario B. In both cases, univariate analysis of the trait with the highest heritability is outperformed by at least one of the MTMM analyses. As a consequence, univariate GWAS and GWAS with the general and contrast MTMM give different rankings of SNPs.

### Selecting candidate genes

A significance threshold of *P *<* *0.0001 was chosen after implementation of genomic control (see below in the section ‘Correction for genomic inflation’). For MTMM this resulted in 43 SNPs meeting this criterion. Such a threshold of 0.0001 is not uncommon in studies involving single‐trait GWAS (e.g. El‐Soda *et al*., 2015; van Rooijen *et al*., 2015; Kooke *et al*., 2016). Given the total number of SNPs analyzed (i.e. 199 589 SNPs having a minor allele frequency > 0.05) and under the null hypothesis of no QTLs and independence of the markers, we arrive at a naive estimate for the expected number of false positives of *c*. 20, which is considerably smaller than the 43 SNPs with *P *<* *0.0001 recorded in the full MTMM, suggesting that about half of the significant SNPs must be true positives. Furthermore, following the procedure described by Benjamini & Hochberg (1995), we estimated the false discovery rate to be 0.45, a number very comparable to our naive estimate earlier. SNPs within a 20 kb region were considered to be part of one linkage disequilibrium (LD) block. This resulted in 30 genomic regions. For presentation purposes, each LD block was represented in figures and heat maps by the SNP with the strongest (absolute) effect, on average, across all traits. For the GWA contrast analyses, the same procedure was followed to define LD blocks and representative SNPs.

### Correcting for genomic inflation

The Wald test is known to suffer from some inflation (Zhou & Stephens, 2014), which we correct for using genomic control (Devlin & Roeder, 1999; Devlin *et al*., 2001), which divides the observed test statistics *T*_1_, …, *T*_*p*_ by the genomic inflation factor. For both the unconstrained MTMM and the MTMM for contrasts described earlier, we observed inflation for small as well as large *P*‐values (i.e. also more *P*‐values close to 1 than expected). Consequently, the usual genomic control procedures based on the observed vs expected median of test statistics gave overly optimistic inflation factors. We therefore applied an alternative genomic control procedure, in which we regress the observed −log_10_(*P*) values on the expected ones, and correct the observed −log_10_(*P*) values for the slope. The genomic inflation factor was 1.24 for the full MTMM, with similar values for the other MTMM analyses (between 1.07 and 1.38). For the full MTMM without correction for population structure (i.e. taking the kinship to be the identity matrix), the inflation factor was 2.36.

## Results

The phenotypic response of a population of 350 *Arabidopsis* accessions to an extensive set of stress‐inducing conditions was quantified *relative to* the respective control treatments. Correcting for the respective control means that in the residual signal for a trait, effects of earliness, flowering time, general robustness, vigor, and so on, have been removed already. Therefore, the traits as analyzed represent a kind of stress *per se* response from which all kinds of disturbances have already been eliminated. Thirty traits, including, for example, root length, number of damaged leaves or number of pathogen‐inflicted spreading lesions (Table 1), were quantified when the plants were exposed to 15 different stresses, that is, four abiotic stresses (drought, salt stress, osmotic stress and heat), seven biotic stresses (parasitic plant, phloem‐feeding aphid, phloem‐feeding whitefly, cell‐content feeding thrips, leaf‐chewing caterpillar, root‐feeding nematode and necrotrophic fungus) and four stress combinations (fungus and caterpillar, drought and fungus, drought and caterpillar, caterpillar and osmotic stress). For detailed information on the carefully standardized stress treatments, the trait definitions and phenotyping, see Supporting Information Methods S1‐S10.

### Heritability of responses to biotic and abiotic stresses

The phenotypic analysis resulted in a wide range of marker‐based, narrow‐sense heritability (Kruijer *et al*., 2015) estimates with 15 traits of low (*h* ^2^ **<* *0.2), 10 of moderate heritability (0.2 < *h* ^2^ **<* *0.5) and five of high (*h* ^2^ > 0.5) heritability (Fig. S1). The number of abiotic stress traits per heritability category was similar, while the number of traits related to biotic and combined stresses decreased with increasing heritability class. The most heritable traits were responses to feeding damage by thrips (Thrips_1; *h* ^2^ **= 0.8), nematodes (*h* ^2^ **= 0.7) and responses to salt (Salt_1 and Salt_3; resp. *h* ^2^ **= 0.6 and *h* ^2^ **= 0.7) and heat (Heat; *h* ^2^ **= 0.6) (Table S1). The traits related to combined stresses have predominantly low heritabilities; however, it should be emphasized that the combined stresses particularly relate to combinations involving fungal and caterpillar stress.

### Genetic commonality underlying responses to different stresses

**Figure 1:**
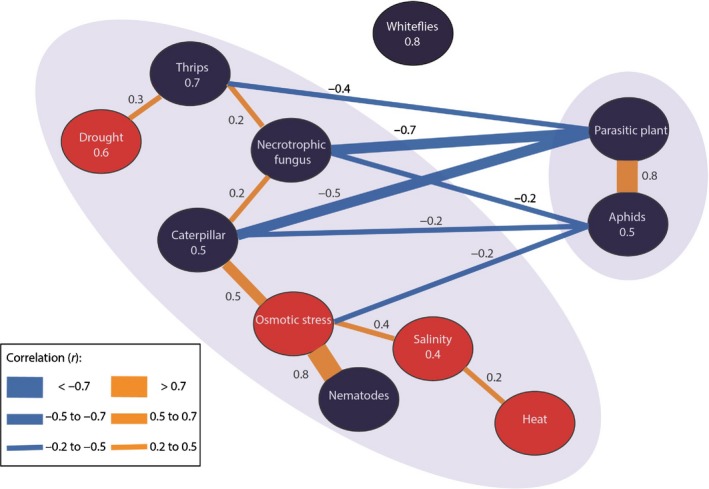
Mean genetic correlations between responses of *Arabidopsis thaliana* to abiotic (red) and biotic (dark blue) plant stresses. The thickness of lines represents the strength of mean genome‐wide correlations, annotated with *r*‐values (orange, positive; blue, negative correlation). The more shared genetic associations between stresses, the higher the absolute genetic correlation. Correlations are negative when alleles have opposite effects, that is, resulting in increased resistance to one stress, but decreased resistance to the other stress. Values in balloons represent mean within‐group correlation (not shown for groups consisting of a single trait). Mean between‐group correlations are not shown if they are below an absolute value of *r *=* *0.2. Two clusters can be distinguished: parasitic plants and aphids; and the other stresses, except whiteflies.

To analyze the phenotypic variation between *Arabidopsis* accessions as a function of molecular marker variation, we used various mixed‐model approaches (see the Materials and Methods section). We estimated marker‐based genetic correlations, that is, correlations based on the genome‐wide commonality of SNP effects underlying pairs of traits (see the Materials and Methods section), to investigate the magnitude of genetic commonality underlying resistance mechanisms in response to a range of biotic and abiotic stresses. For brevity, we will refer to these marker‐based genetic correlations as genetic correlations. Such genetic correlations can be interpreted as upper bounds to the joint determination of pairs of traits by genetic factors. Genetic correlation analysis revealed a strong connection between the responses to parasitic plants and to aphids (*r *=* *0.8), which were both negatively associated with other stress responses (Fig. 1). Parasitic plants and aphids have in common that they target phloem and xylem tissue (Tjallingii & Hogen Esch, 1993; Dorr & Kollmann, 1995), and induce the SA phytohormonal pathway (De Vos *et al*., 2005; Runyon *et al*., 2008). By contrast, the biotic stress responses that were negatively associated with the responses to parasitic plants and aphids, that is, responses to necrotrophic fungi, caterpillars, and thrips, represent JA‐inducing stresses (De Vos *et al*., 2005; Pieterse *et al*., 2009, 2012). Because the SA and JA pathways predominantly interact through negative crosstalk (Pieterse *et al*., 2009), the two main clusters resulting from the genetic correlation analysis represent different phytohormonal signaling response mechanisms. We also observed a strong genetic correlation between plant responses to osmotic stress and root‐feeding nematodes. This supports the notion that root‐knot nematodes trigger a differentiation of root cells to multinucleate giant cells with severely altered water potential and osmotic pressure (Baldacci‐Cresp *et al*., 2015). While the correlations between traits at the phenotypic level were generally rather low, the genetic correlation analysis revealed a common genetic basis underlying the responses to sets of single and combined stresses (Fig. S2).

### Candidate genes underlying responses to stresses

**Figure 2:**
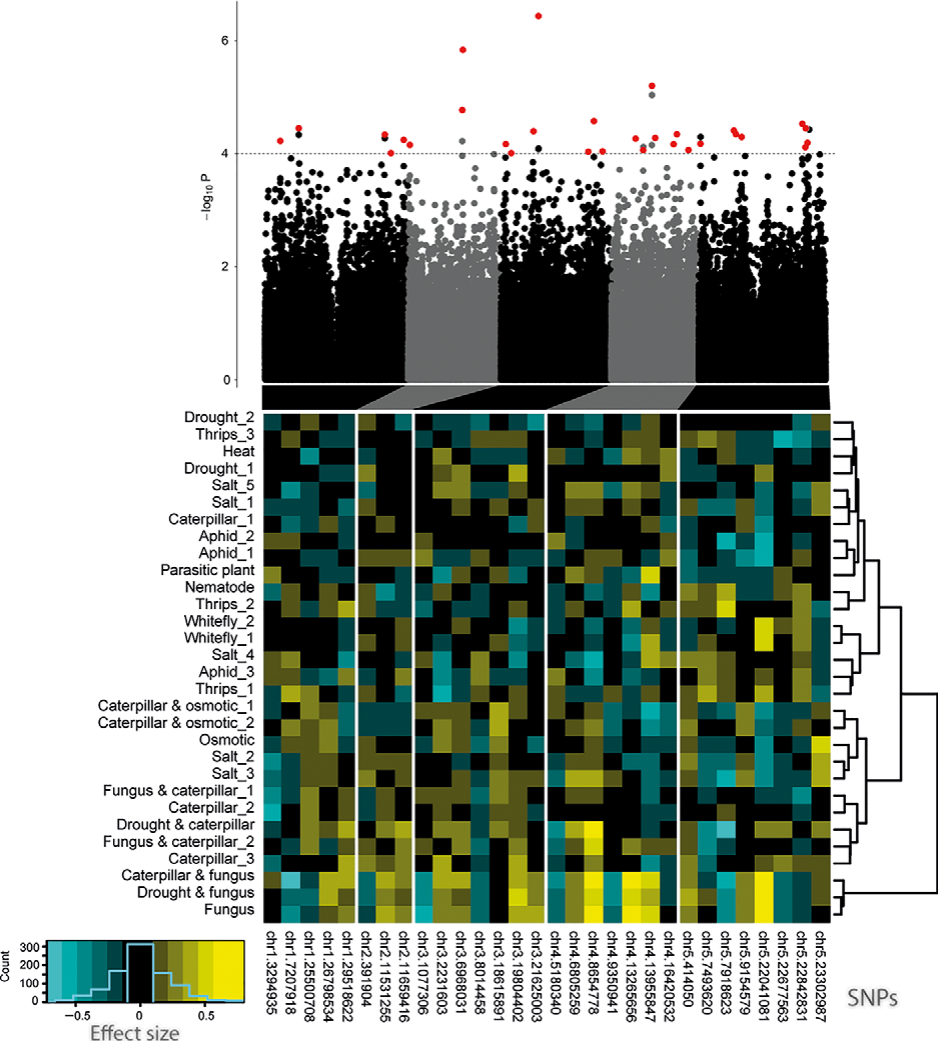
Multi‐trait mixed‐model (MTMM) genome‐wide association (GWA) mapping with 30 different stress responses of *Arabidopsis thaliana*. The top panel shows the 214 000 single nucleotide polymorphisms (SNPs) with their corresponding −log_10_(*P*) values for the five chromosomes. The lower panel depicts the trait‐specific effect sizes of the rare alleles for significant SNPs (*P *<* *0.0001) as estimated by the full MTMM. When several SNPs were located within the 20 kb linkage disequilibrium half‐windows around the most significant SNP in a region, the effects for the SNP with the strongest absolute average effects are shown (red‐flagged in the Manhattan plot). SNPs are named by chromosome number and position on the chromosome. Negative effect sizes (blue) correspond to reduced plant resistance as a result of the rare allele, and positive effect sizes (yellow) correspond to increased resistance as a result of the rare allele. Stress responses were clustered hierarchically according to their effect, using Ward's minimum variance method. The key shows the frequency distribution for the effect sizes of the SNPs.

To identify individual candidate genes that contributed most to the pattern of genetic correlations, we fitted multi‐trait QTL mixed models (MTMMs) to the total set of 30 traits, using a 214 000 SNP set that is commonly used for GWAS in Arabidopsis (Kim *et al*., 2007; Atwell *et al*., 2010; Li *et al*., 2010; Horton *et al*., 2012; Bac‐Molenaar *et al*., 2015). Our multi‐trait GWA approach closely follows the modeling framework developed by Zhou & Stephens (2014) and generalizes the use of MTMMs as described previously (Boer *et al*., 2007; Malosetti *et al*., 2008; Alimi *et al*., 2013) for classical biparental offspring populations to association panels. This GWA analysis identified 30 chromosome regions with multiple, significant SNP–trait associations. From each of those regions, the significant SNP with the strongest effect was chosen to represent the locus (Fig. 2; Table S2). Clustering of stresses by estimated SNP‐effect profiles (Fig. 2) indicates that multiple SNPs were associated with response to more than one stress. Stress combinations induced large QTL allele substitution effects in the MTMM mapping (Fig. 2; Table S2), indicating that combinations of stresses trigger broad‐spectrum defensive mechanisms. A total of 125 genes were in LD with the 30 most significant SNPs from the GWA analysis. Twenty of these genes were stress‐related according to gene ontology annotation data (Table S3). Of these 20 genes, six have been functionally characterized by at least one study (Table 2a). For these six genes, we explored expression data to evaluate the biological relevance of these genes in stress‐responsive mechanisms of *Arabidopsis* (Fig. S3). Of special interest were SNPs chr5.7493620, chr5.22041081 and chr4.6805259, which were in LD with *WRKY38* (encoding a WRKY transcription factor involved in SA‐dependent disease resistance) (Kim *et al*., 2008), *AtCNGC4* (involved in pathogen resistance) (Chin *et al*., 2013) and *RMG1* (coding for disease resistance protein) (Yu *et al*., 2013), respectively.

**Table 2:** Candidate *Arabidopsis thaliana* genes resulting from (a) multi‐trait mixed‐model (MTMM) analysis of all 30 stress responses as presented in Fig. 2; and (b) contrast‐specific analysis with MTMM for contrasting effects of biotic and abiotic stresses as presented in Fig. 3

Marker^a^	Gene in LD	Gene name	Gene description^b^	Responsiveness	References
(a)					
chr2.11659416	*AT2G27250*	*CLV3*	One of the three *CLAVATA* genes controlling the size of the shoot apical meristem (SAM) in *Arabidopsis*	Unknown	Clark *et al*. (1996); Fletcher *et al*. (1999); Shinohara & Matsubayashi (2010)
chr3.19804402	*AT3G53420*	*PIP2*	A member of the plasma membrane intrinsic protein subfamily PIP2	Heat, salt and heat, heat and silwet	Martiniere *et al*. (2012); Peret *et al*. (2012); Rasmussen *et al*. (2013); Sanchez‐Romera *et al*. (2014)
chr4.6805259	*AT4G11170*	*RMG1*	Encodes RMG1 (Resistance Methylated Gene 1), an NB‐LRR disease resistance protein with a Toll/interleukin‐1 receptor (TIR) domain at its N terminus	Flagellin	Yu *et al*. (2013)
chr5.7493620	*AT5G22570*	*WRKY38*	Member of WRKY Transcription Factor; Group III	SA, *Pseudomonas*	Mare *et al*. (2004); Kim *et al*. (2008)
chr5.22041081	*AT5G54250*	*CNGC4*	Member of cyclic nucleotide gated channel family, a downstream component of the signaling pathways leading to hypersensitive response (HR) resistance. Mutant plants exhibit gene‐for‐gene disease resistance against avirulent *Pseudomonas syringae* despite the near‐complete absence of the HR. Salicylic acid accumulation in *dnd2* mutants is completely *PAD4*‐independent	Cold, flagellin	Jurkowski *et al*. (2004); Keisa *et al*. (2011); Chin *et al*. (2013); Rasmussen *et al*. (2013)
chr5.23302987	*AT5G57560*	*TCH4*	Encodes a cell wall modifying enzyme, rapidly up‐regulated in response to environmental stimuli	Heat, heat and silwet, heat and salt, heat and high light, high light, high light and cold, high light and salt	Braam & Davis (1990); Xu *et al*. (1996); Purugganan *et al*. (1997); Iliev *et al*. (2002); Rasmussen *et al*. (2013)
(b)					
chr1.30381439	*AT1G80820*	*CCR2*	*CINNAMOYL COA REDUCTASE*. Encodes a cinnamoyl CoA reductase isoform. Involved in lignin biosynthesis	Cold and flagellin and silwet	Luderitz & Grisebach (1981); Lauvergeat *et al*. (2001); Zhou *et al*. (2010); Rasmussen *et al*. (2013)
chr1.30381439	*AT1G80840*	*WRKY40*	Pathogen‐induced transcription factor. Binds W‐box sequences *in vitro*. Forms protein complexes with itself and with WRKY60. Coexpression with *WRKY18* or *WRKY60* made plants more susceptible to both *P. syringae* and *Botrytis*	Cold and flagellin and silwet	Chen *et al*. (2010a); Pandey *et al*. (2010); Liu *et al*. (2012); Rasmussen *et al*. (2013)
chr1.6038270	*AT1G17610*	*CHS1*	*CHILLING SENSITIVE 1*, mutant accumulates steryl‐esters at low temperature	Cold and high light	Rasmussen *et al*. (2013); Wang *et al*. (2013); Zbierzak *et al*. (2013)
chr5.171177	*AT5G17640*	*ASG1*	*ABIOTIC STRESS GENE 1*; expression of this gene is induced by ABA and salt stress	ABA, salt	Coste *et al*. (2008); Batelli *et al*. (2012)
chr5.23247572	*AT5G57380*	*VIN3*	Encodes a plant homeodomain protein *VERNALIZATION INSENSITIVE 3* (*VIN3*). *In planta* VIN3 and VRN2, VERNALIZATION 2, are part of a large protein complex that can include the polycomb group (PcG) proteins *FERTILIZATION INDEPENDENT ENDOSPERM* (*FIE*), *CURLY LEAF* (*CLF*), and *SWINGER* (*SWN* or *EZA1*). The complex has a role in establishing *FLC* (*FLOWERING LOCUS C*) repression during vernalization	Cold	Sung *et al*. (2007); Bond *et al*. (2009); Finnegan *et al*. (2011)
chr5.23293119	*AT5G57560*	*TCH 4*	Encodes a cell wall‐modifying enzyme	Heat, heat and silwet, heat and salt, heat and high light, high light, high light and cold, high light and salt	Braam & Davis (1990); Xu *et al*. (1996); Purugganan *et al*. (1997); Iliev *et al*. (2002); Rasmussen *et al*. (2013)
chr5.23293870	*AT5G57490*	*VDAC4*	Encodes a voltage‐dependent anion channel (VDAC: AT3G01280/VDAC1)	*Pseudomonas*	Lee *et al*. (2009); Tateda *et al*. (2011)
chr5.23366252	*AT5G57685*	*GDU3*	Encodes a member of the GDU (glutamine dumper) family proteins involved in amino acid export: At4g31730 (GDU1)	Unknown	Chen *et al*. (2010b)

NB‐LRR, nucleotide binding site–leucine‐rich repeat.

a Markers derived from MTMM analysis (see Fig. 2).

b Based on information on http://www.arabidopsis.org/tools/bulk/go/index.jsp.

### Phytohormonal signaling underlying contrasts in stress responses

The MTMM framework allowed constraints to be imposed on the values of the estimated QTL effects, thereby providing a powerful testing framework for QTLs that have a common effect for the stresses belonging to one particular group of stresses, as contrasted with the effect for another group of stresses (see the Materials and Methods section ‘Multi‐trait GWAS’). We investigated whether polymorphisms for genes involved in SA and JA biosynthesis or genes responsive to signals from these pathways were the cause of the negative genetic correlations between the groups of traits sharing one or the other phytohormonal signaling pathway. To this end, we performed multi‐trait GWA mapping to test the contrast between parasitic plant and aphid response vs the most negatively correlated traits, that is, fungus, caterpillar, thrips and drought response (Fig. 1). Fifteen SNPs were significantly associated with contrasting effects between the two trait clusters (Fig. S4). Seven of these SNPs were in LD with one or more genes known to be involved in JA‐, SA‐ or resistance‐related signal transduction (Table S4). Among these genes are *LOX5*, whose product is involved in facilitating aphid feeding (Nalam *et al*., 2012a,b), *MYB107* encoding a transcription factor responsive to SA (Stracke *et al*., 2001; Chen *et al*., 2006), the JA‐inducible genes *TPS02* and *TPS03* encoding terpene synthases (Huang *et al*., 2010), and *MES16*, encoding a methyl jasmonate esterase (Christ *et al*., 2012). Using TAIR10 annotations, we found that in total there are 371 genes that have an annotation related to JA and SA signaling (JA‐SA genes). Our GWA analysis identified significant SNPs inside or in a 20 kb neighborhood of five of those. In the remainder of the genome (i.e. non JA‐SA), we identified 162 genes close to or with significant SNPs. So, in candidate regions for JA‐SA, we had a ratio of 5/371 = 1.35% significant genes, while in noncandidate regions, we found 162/27863 = 0.58%. This is an enrichment of 2.33 times, significant at *α *= 0.05 (Fisher's exact probability test, mid‐*P* value* *<* *0.046; Rivals *et al*., 2007). Following Atwell *et al*. (2010), an upper bound for the false discovery rate is then 1/2.33 = 0.43.

In addition to screening for SNPs with contrasting effects, we screened for SNPs with a similar effect across the earlier‐mentioned trait clusters (Fig. S5) and found candidate genes involved in oxidative stress and plant responses to salinity and pathogens (Table S5).

### QTLs underlying contrasts in responses to biotic and abiotic stresses

**Figure 3:**
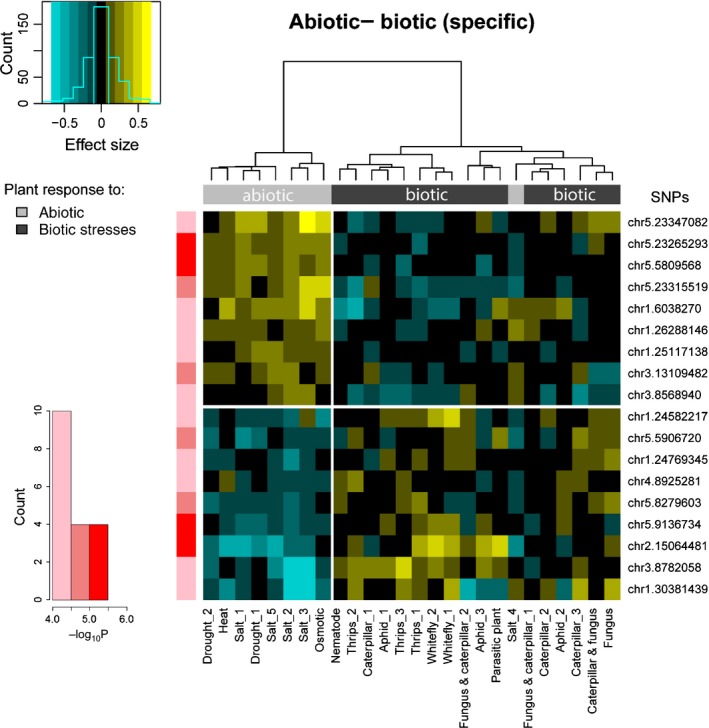
Genetic associations specific for contrasting responses of *Arabidopsis thaliana* to abiotic and biotic stresses. Genetic associations (in red) were estimated with a contrast‐specific genome‐wide association analysis using a multi‐trait mixed model (MTMM). For exploratory purposes, significant single nucleotide polymorphisms (SNPs) (*P *≤ 10^−4^) for the biotic–abiotic contrast were clustered on their trait‐specific effect sizes as estimated in the full MTMM, that is, without imposing a contrast restriction on the SNP effects. If there was another SNP in LD that had a higher effect size, this SNP was used as a representative of the LD block. Negative effects (blue) were cases where the rare allele was associated with a detrimental effect on the plants, while positive effects (yellow) were cases where the rare allele was associated with increased resistance to the stress. The rare alleles of the top nine SNPs are associated with enhanced resistance to abiotic stresses and reduced resistance to biotic stresses; the bottom nine SNPs show the inverse. Stresses were clustered on the basis of SNP effects using Ward's minimum variance method. The key shows the frequency distribution of SNPs across effect sizes.

We expected a negative correlation between the responses to abiotic and biotic stresses as a result of antagonistic interactions between ABA and the SA and JA‐ET pathways (Anderson *et al*., 2004; Fujita *et al*., 2006; De Torres Zabala *et al*., 2009; Kissoudis *et al*., 2015). Testing for this contrast within the GWA analysis using our MTMM approach significantly identified 43 SNPs with a QTL effect that changed sign between biotic and abiotic conditions. For presentation purposes, traits were grouped by a cluster analysis across SNPs, while SNPs were grouped by clustering across traits. Fig. 3 shows the SNPs with the strongest overall effects, identified in 18 LD intervals. The minor alleles of nine of these SNPs displayed a positive effect on biotic stress response traits and a negative effect on abiotic response traits. The remaining nine SNPs displayed the opposite effect (Fig. 3). Several candidate genes were identified in LD with the SNPs that are specific for plant responses to either abiotic or biotic stresses (Table 2b), such as *TCH4* (encoding a cell wall‐modifying enzyme)*, AtCCR2* (involvement in lignin biosynthesis) and *ASG1* (a gene induced by ABA and salt stress). Transcription data (Fig. S6) support the notion that these genes play a contrasting role in responses to abiotic and biotic stresses and reveal an antagonistic responsiveness between ABA and JA treatment (*TCH4*) or a specific responsiveness to either ABA (*AtCCR2*,* ASG1*,* ATVDAC4*) or JA (*ATWRKY40*). This is in line with the hypothesis that there are antagonistic effects between abiotic stress responses, predominantly involving the ABA pathway, and wound and biotic stress responses involving the JA‐ET or SA pathways (Kissoudis *et al*., 2015). Previous studies have, however, also revealed an overlap in abiotic and biotic plant responses, such as similar transcriptomic perturbations after salinity and pathogen stress (Ma *et al*., 2006). A screen for QTLs with similar effects on resistance to biotic and abiotic stress (Fig. S7) identified three genes annotated to be responsive to stress stimuli (Table S6). Transcriptional data show that these genes respond differentially to different (a)biotic stresses and phytohormones (Fig. S8). *ARGAH2*, encoding an arginase enzyme with a role in the metabolism of polyamines and nitric oxide, is involved in both SA‐ and JA‐mediated resistance to both biotrophic and necrotrophic pathogens, and is also responsive to abiotic stimuli such as temperature, salt and light intensity (Fig. S8) (Jubault *et al*., 2008; Gravot *et al*., 2012; Rasmussen *et al*., 2013). *PKS1* is known to be involved in adaptation in plant growth in response to light (Fankhauser *et al*., 1999; Molas & Kiss, 2008), but also seems to be responsive to *Botrytis* (Fig. S8). These genes are promising candidates for consistent effects across biotic and abiotic stresses.

### QTLs underlying contrasts in responses to below‐ and above‐ground stresses

We expected a negative correlation between responses to below‐ and above‐ground stresses. A strong QTL signal was found on chromosome 1 for this contrasting response (Fig. S9). The associated marker (chr1.13729757) had 12 genes in LD with it, of which 11 are annotated as pseudogenes. Transcriptional data on abiotic stresses for the only protein coding gene (*AT1G36510*) show an up‐regulation in above‐ground tissues, yet a down‐regulation in the root tissues (Winter *et al*., 2007). Marker chr5.16012837 showed the strongest signal for similar effects on responses to below‐ and above‐ground stresses (Fig. S10) for which the *pathogenesis‐related thaumatin superfamily protein* (*AT5G40020*) is the most promising candidate gene.

### Validation of identified QTLs

**Figure 4:**
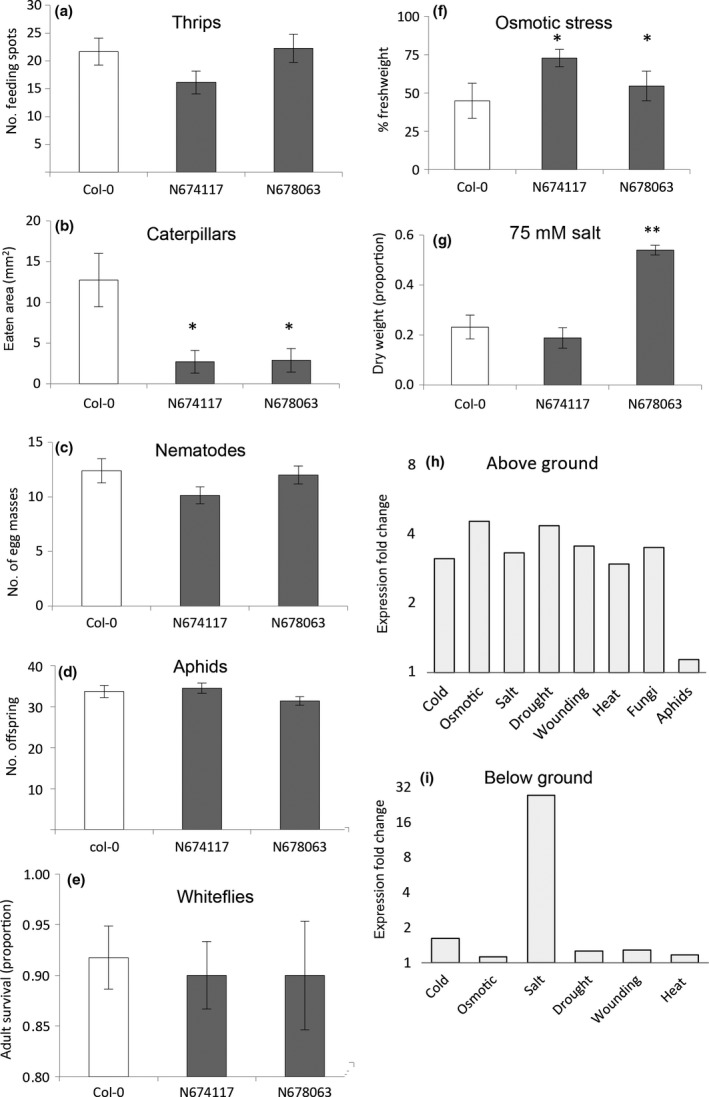
Phenotypes of *RMG1* T‐DNA mutant screenings for *Arabidopsis thaliana*. Phenotypes are given for two T‐DNA lines in the *RMG1* gene and for Col‐0 as control. (a) Number of thrips feeding spots on a detached leaf at 6 d postinfestation (*n *=* *24). (b) Leaf area consumed by *Pieris rapae* caterpillars (*n *=* *6). (c) Number of nematode egg masses (*n *=* *23). (d) Number of *Myzus persicae* aphid offspring (*n *=* *10–17). (e) Percentage survival of adult whiteflies (*Aleyrodes proletella*) (*n *=* *10). (f) Plant FW after osmotic treatment in comparison with control (% relative to control) (*n *=* *4). (g) Plant DW after 75 mM salt treatment in comparison with control (ratio) (*n *=* *7–10); mean ± SE; *, *P *<* *0.05; **, *P *<* *0.01 (difference in comparison with Col‐0). (h, i) Relative expression fold‐change for *RMG1* compared with untreated control plants in above‐ground (h) and below‐ground (i) tissue. Expression data from *Arabidopsis* eFP browser (http://bbc.botany.utoronto.ca).

To obtain experimental support for the most interesting QTLs resulting from the MTMM, we tested homozygous T‐DNA insertion lines for candidate genes *RMG1* and *WRKY38* (both resulting from the MTMM analysis), and *TCH4* (from MTMM analysis on biotic vs abiotic contrast) for several of the stresses addressed in this study. Two independent *rmg1* T‐DNA insertion lines showed a phenotype that was different from the wild‐type (Col‐0) for some of the stress conditions (Fig. 4; Methods S11), being more resistant to caterpillar feeding and osmotic stress (Fig. 4). *RMG1* (AT4G11170) encodes a nucleotide binding site–leucine‐rich repeat disease resistance protein, which acts as a pattern‐recognition receptor that recognizes evolutionarily conserved pathogen‐derived signatures, and transcription is induced by the bacterial peptide flg22 (Yu *et al*., 2013). The rare allele of the corresponding marker chr4.6805259 is associated with enhanced resistance to salt stress and the combined stresses ‘caterpillar and drought’ and ‘caterpillar and fungus’ and with enhanced susceptibility to drought stress. Gene expression data show that *RMG1* is up‐regulated by several abiotic and biotic stresses (Fig. 4). In addition, gene ontology enrichment analysis of the coexpression network of *RMG1* shows an overrepresentation of genes involved in immune responses and maintenance of ion homeostasis. The latter is based upon coexpression with five genes encoding glutamate receptors (*GLR1.2*,* GLR1*.*3*,* GLR2.5*,* GLR2.8*, and *GLR2.9*), putatively involved in ion‐influx‐mediated long‐distance signaling of wound, pathogen and salt stress (Ma *et al*., 2006; Mousavi *et al*., 2013; Choi *et al*., 2014; Kissoudis *et al*., 2015). T‐DNA insertion lines for *TCH4* and *WRKY38* did not show a phenotype different from the wild‐type (Col‐0) for any of the tested stress conditions. Whether this is dependent on the genetic background used remains to be investigated.

Summarizing, our multi‐trait GWA methodology facilitated a detailed analysis of the genetic architecture of resistance in *Arabidopsis* to a wide diversity of biotic and abiotic stresses. Application of this methodology revealed novel candidate genes associated with multiple stress responses, where specific contrasts were identified with some genes positively associated with the resistance to one set of stresses while being negatively associated with another set of stresses. In plant breeding (Brady *et al*., 2005; Ballesteros *et al*., 2015), such genes are classified as adaptive. Alternatively, other genes were identified with consistent effects across a wide spectrum of stress conditions. Such genes are labeled as constitutive in the plant breeding literature (Brady *et al*., 2005; Ballesteros *et al*., 2015). Both adaptive and constitutive QTLs are important factors to contribute to improved stress resistance and tolerance in commercial crop species (Brady *et al*., 2005; Ballesteros *et al*., 2015).

## Discussion

We developed a novel mixed‐model approach to multi‐trait GWA mapping with a special feature for testing contrasts between groups of stresses to identify the genetic architecture underlying a total of 30 stress response traits in Arabidopsis. The strength of our statistical approach was that our multi‐trait mixed model accounted simultaneously for dependencies between genotypes and between traits, providing a natural and appropriate correction for multiple testing, while maximizing power for the detection of QTLs for the stress contrast under study. As we addressed a large number of stresses, our phenotyping experiments were distributed across a series of laboratories and were not performed simultaneously. To mitigate as much as possible the occurrence of QTLs induced purely by experiment‐specific differences in plant management and environmental control, our phenotypic responses were defined in terms of control‐corrected responses. This type of correction will emphasize QTLs for resistance and tolerance *per se* and will decrease detection power for QTLs related to development and viability.

The extensive phenotyping executed in this study was done under carefully controlled conditions in climate chambers. Ideally, phenotyping should be done in nature because that is where genetic variation is exposed to natural selection (Bergelson & Roux, 2010; Brachi *et al*., 2010, 2013). Here, we have phenotyped the plant population to 15 different stresses under laboratory conditions and our data show an interesting pattern based on genetic correlations that matches with phytohormonal signaling underlying stress responses (Fig. 1). This indicates that the genetic architecture recorded here is biologically relevant. Drought and salt stress responses share signal transduction mechanisms (Zhu, 2002) which are represented by the genetic correlations recorded (Fig. 1). Insect damage is commonly associated with drought or osmotic stress and this is also clear from overlap in underlying phytohormonal signalling (Pieterse *et al*., 2012). Fig. 1 shows that drought stress and osmotic stress correlate with insect stresses. Extending studies of genetic variation and the genetic architecture underlying responses to multiple stresses to natural conditions will be an important next step (Bergelson & Roux, 2010).

Through the approach developed here, candidate genes for stress responses were identified that are involved in contrasting responses when comparing biotic and abiotic stresses, above‐ and below‐ground stresses, and attack by phloem feeders vs other biotic stresses. Among these genes many are involved in phytohormone‐mediated processes, supporting the notion that the phytohormonal regulatory network plays an important role in plant stress responses (Pieterse *et al*., 2012). The MTMM approach further showed that certain SNPs were associated with multiple stress responses and that transcriptional patterns of genes to which the SNPs were linked, as well as the phenotype expressed upon knocking out one of these genes, matched the observed stress responses of the plants. The *RMG1* gene that was identified through this procedure has relevant effects on plant phenotype in the context of responses to individual stresses. *RMG1* is a bacterium‐inducible resistance gene whose activity is modulated by the plant through RNA‐directed DNA methylation (RdDM) (Yu *et al*., 2013). *RMG1* expression activates the SA pathway (Yu *et al*., 2013). Thus, the increased resistance against caterpillars in *rmg1* mutants may be the result of elimination of SA‐mediated interference with JA‐induced resistance to caterpillars (Pieterse *et al*., 2012). *RMG1* appears to be inducible by several stresses and deserves further in‐depth analysis for its role in plant response to multiple stresses. Our data show that for the 30 most significant SNPs resulting from the MTMM analysis, the average absolute effect size for double stresses is higher, on average, than that for single stresses (*P *<* *0.007, Table S2). This suggests that resistance mechanisms involved in countering dual stresses are of a more general nature, in contrast to the rather specific resistance mechanisms involved in single stress responses. However, the combined stresses included in this study particularly involve fungal and caterpillar stresses. Future studies including other combined stresses are needed to further investigate the suggested pattern.

The MTMM framework that we used for GWA mapping provides unbiased estimates for QTL allele substitution effects together with correct standard errors for these effects. Within the same framework we developed unique facilities to test hypotheses on QTL × stress interactions in multi‐trait models, which are not available in competing meta‐analysis approaches (Zhu *et al*., 2015). The variance‐covariance structure that we used for the polygenic term protects against inflated type I error, that is, too many false‐positive SNP–trait associations, as a consequence of population structure and kinship on the genotypic side and genetic correlations between traits on the trait side. The inclusion of trait correlations will, for most QTLs, improve the power of detection in comparison to single‐trait GWA mapping (Korte *et al*., 2012; Zhou & Stephens, 2014; see ‘Multi‐trait GWAS’ in the Materials and Methods section). For a comparison of the MTMM analysis with single‐trait analyses, see ‘Simulations to compare power for full MTMM, contrast MTMM and univariate analysis’ in the Materials and Methods section, Methods S12 and Figs S11 and S12. Our choice for the variance‐covariance structure of the polygenic term as a Kronecker product of a compressed kinship on the genotypes with an approximated unstructured variance‐covariance model on the environments is sometimes used in plant breeding for genomic prediction models (Burgueno *et al*., 2012). However, implementation of such models in GWA mapping and especially on the scale that we present here, with 30 traits, is unprecedented and is practically far from straightforward. It required substantial work on preparatory phenotypic analyses as well as fine‐tuning of the genotypic and trait variance‐covariance structures to achieve convergence of the mixed models.

The MTMM analyses identified candidate genes associated with contrasting responses to biotic and abiotic stresses. Stress combinations appeared to have a strong influence on the MTMM outcome, indicative for significant interactions between different stresses when occurring simultaneously, and underlining the importance of studying the resistance of plants to combinations of stress. Transcriptional data and phenotyping of mutants provide initial support for the role of several of the candidate genes identified. Studies of plant responses to a diverse set of biotic stresses show that the transcriptional pattern is stress‐specific and that phytohormonal signaling pathways can explain up to 70% of the induced gene regulation (De Vos *et al*., 2005). Taking the outcome of the MTMM analyses to investigate the involvement of identified candidate genes in the resistance of plants to several stresses, not only in Arabidopsis but also in related crop species, such as, for example, *Brassica* species, will be valuable in the breeding by design of future crops to protect them against combinations of stresses, including biotic and abiotic stresses. This will be of great value for next‐generation crops.

## Author contributions

M.P.M.T., N.H.D.O., K.J.K., S.C., P‐P.H., J.A.B‐M., C.B., J. Bucher, J.B‐L., X.C., E.F.F., M.M.J., W.L., J.A.v.P., S.W. and G.L.W. phenotyped the plants; M.P.M.T., N.H.D.O., K.J.K., S.C., P‐P.H., M.G.M.A., J.A.B‐M., J. Bakker, H.J.B., J. Bucher, C.B., X.C., E.F.F., M.A.J., M.M.J., J.J.B.K., W.L., C.M.J.P., C.R‐S., G.S., C.T., J.J.A.v.L., J.A.v.P., C.C.v.S., S.C.M.v.W., R.G.F.V., R.V., B.V., D.V., S.W., G.L.W. and M.D. designed the phenotyping experiments and made initial analyses; W.K., J.v.H. and F.A.v.E. developed the multi‐trait mixed model; W.K., J.v.H., M.P.M.T., N.H.D.O., K.J.K., S.C., P‐P.H., B.U., F.A.v.E. and M.D. analyzed the total dataset. M.D. and F.A.v.E. coordinated the study as a whole. M.P.M.T., N.H.D.O., K.J.K., S.C., P‐P.H., W.K., J.v.H., F.A.v.E. and M.D. wrote the manuscript with input from J.J.B.K., C.J.M.P. and M.G.M.A. and all authors proofread the final version of the manuscript.

## Supporting information

Please note: Wiley Blackwell are not responsible for the content or functionality of any Supporting Information supplied by the authors. Any queries (other than missing material) should be directed to the *New Phytologist* Central Office.

**Fig. S1** Narrow‐sense heritability for *Arabidopsis thaliana* resistance to abiotic and biotic stresses.
**Fig. S2** Genetic and phenotypic correlation matrix.
**Fig. S3** Expression data of six candidate genes (resulting from MTMM) in plants exposed to biotic or abiotic stress factors, relative to control conditions.
**Fig. S4** Genetic associations specific for plant responses to the main clusters of the genetic correlation network (see Fig. 1): parasitic plant and aphid vs fungus, caterpillar, thrips and drought.
**Fig. S5** Genetic associations common for plant response to the main clusters of the genetic correlation network: parasitic plant and aphid, on the one hand, vs fungus, caterpillar, thrips and drought on the other.
**Fig. S6** Expression data of six candidate genes (resulting from MTMM analysis) in plants exposed to biotic or abiotic stress factors, relative to control conditions.
**Fig. S7** Genetic associations common for plant responses to abiotic and biotic stresses.
**Fig. S8** Expression data of three candidate genes (resulting from MTMM) in plants exposed to biotic or abiotic stress factors, relative to control conditions.
**Fig. S9** Genetic associations specific for plant responses to either below‐ or above‐ground stress.
**Fig. S10** Genetic associations common for plant responses to below‐ and above‐ground stresses.
**Fig. S11** Power of MTMM in simulations.
**Fig. S12** Comparison of SNPs identified by MTMM and univariate GWAS.
**Table S1** Data overview on phenotyping the 350 *Arabidopsis thaliana* accessions of the HapMap collection
**Table S2** Summed effect sizes of 30 most significant SNPs in MTMM per trait
**Table S3** 125 candidate genes derived from the Multitrait Mixed Model analysis
**Table S4** Genes in linkage with SNPs with –log_10_(*P*) score > 4 (20 kb half‐window size) in the contrast‐specific GWA mapping of parasitic plants and aphids, on the one hand, vs fungus, caterpillar, thrips and drought on the other
**Table S5** Candidate genes in linkage with SNPs with –log_10_(*P*) score > 4 (20 kb half‐window size) that have common effects on plant response to parasitic plants and aphids, on the one hand, vs fungus, caterpillar, thrips and drought on the other
**Table S6** Candidate genes in linkage with SNPs with –log_10_(*P*) score > 4 (20 kb half‐window size) that have common effects on biotic and abiotic stress responses
**Methods S1** Salt.
**Methods S2** Abiotic.
**Methods S3** Caterpillar – combinatory stress.
**Methods S4** Parasitic plants.
**Methods S5** Nematodes.
**Methods S6** Whiteflies.
**Methods S7** Aphids.
**Methods S8** Thrips.
**Methods S9** Drought – combinatory stress.
**Methods S10** Fungus – combinatory stress.
**Methods S11** Screening of T‐DNA lines.
**Methods S12** Simulations to compare power for full MTMM, contrast MTMM and univariate analysis.
