# Supplementary material for: Genetic architecture of plant stress resistance: multi‐trait genome‐wide association mapping
Source: New Phytol. 2016 Oct 4;213(3):1346–62. doi: 10.1111/nph.14220 (PMC5248600; doi:10.1111/nph.14220)
Supplement: Supplementary file 1 — Fig. S1 Narrow‐sense heritability for Arabidopsis thaliana resistance to abiotic and biotic stresses. Fig. S2 Genetic and phenotypic correlation matrix. Fig. S3 Expression data of six candidate genes (resulting from MTMM) in plants exposed to biotic or abiotic stress factors, relative to control conditions. Fig. S4 Genetic associations specific for plant responses to the main clusters of the genetic correlation network (see Fig. 1): parasitic plant and aphid vs fungus, caterpillar, thrips and drought. Fig. S5 Genetic associations common for plant response to the main clusters of the genetic correlation network: parasitic plant and aphid, on the one hand, vs fungus, caterpillar, thrips and drought on the other. Fig. S6 Expression data of six candidate genes (resulting from MTMM analysis) in plants exposed to biotic or abiotic stress factors, relative to control conditions. Fig. S7 Genetic associations common for plant responses to abiotic and biotic stresses. Fig. S8 Expression data of three candidate genes (resulting from MTMM) in plants exposed to biotic or abiotic stress factors, relative to control conditions. Fig. S9 Genetic associations specific for plant responses to either below‐ or above‐ground stress. Fig. S10 Genetic associations common for plant responses to below‐ and above‐ground stresses. Fig. S11 Power of MTMM in simulations. Fig. S12 Comparison of SNPs identified by MTMM and univariate GWAS. Table S1 Data overview on phenotyping the 350 Arabidopsis thaliana accessions of the HapMap collection Table S2 Summed effect sizes of 30 most significant SNPs in MTMM per trait Table S3 125 candidate genes derived from the Multitrait Mixed Model analysis Table S4 Genes in linkage with SNPs with –log10(P) score > 4 (20 kb half‐window size) in the contrast‐specific GWA mapping of parasitic plants and aphids, on the one hand, vs fungus, caterpillar, thrips and drought on the other Table S5 Candidate genes in linkage with SNPs with –log10(P) score > 4 (20 kb half‐wi [file NPH-213-1346-s001.pdf]

New Phytologist

New Phytologist **Supporting Information Figs S1–S12, Tables S1–S6 and Methods S1–S12**

Article title: Genetic architecture of plant stress resistance: multi-trait genome-wide association mapping

Authors: Manus P.M. Thoen, Nelson H. Davila Olivas, Karen J. Kloth, Silvia Coolen, Ping-Ping Huang, Mark G. M. Aarts, Johanna A. Bac-Molenaar, Jaap Bakker, Harro J. Bouwmeester, Colette Broekgaarden, Johan Bucher, Jacqueline Busscher-Lange, Xi Cheng, Emilie F. Fradin, Maarten A. Jongsma, Magdalena M. Julkowska, Joost J. B. Keurentjes, Wilco Ligterink, Corné M. J. Pieterse, Carolien Ruyter-Spira, Geert Smant, Christa Testerink, Björn Usadel, Joop. J. A. van Loon, Johan A. van Pelt, Casper C. van Schaik, Saskia C. M. van Wees, Richard G. F. Visser, Roeland Voorrips, Ben Vosman, Dick Vreugdenhil, Sonja Warmerdam, Gerrie L. Wiegers, Joost van Heerwaarden, Willem Kruijer, Fred A. van Eeuwijk and Marcel Dicke

Article acceptance date: 17 August 2016

The following Supporting Information is available for this article:

Fig. S1 Narrow sense heritability for *Arabidopsis thaliana* resistance to abiotic and biotic stresses.

Fig. S2 Genetic and phenotypic correlation matrix.

Fig. S3 Expression data of six candidate genes (resulting from MTMM) in plants exposed to biotic or abiotic stress factors, relative to control conditions.

Fig. S4 Genetic associations specific for plant responses to the main clusters of the genetic correlation network (see Fig. 1): parasitic plant and aphid vs fungus, caterpillar, thrips and drought.

Fig. S5 Genetic associations common for plant response to the main clusters of the genetic correlation network: parasitic plant and aphid on the one hand vs fungus, caterpillar, thrips and drought on the other hand.

Fig. S6 Expression data of six candidate genes (resulting from MTMM analysis) in plants exposed to biotic or abiotic stress factors, relative to control conditions.

Fig. S7 Genetic associations common for plant responses to abiotic and biotic stresses.

Fig. S8 Expression data of three candidate genes (resulting from MTMM) in plants exposed to biotic or abiotic stress factors, relative to control conditions.

Fig. S9 Genetic associations specific for plant responses to either below- or aboveground stress.

Fig. S10 Genetic associations common for plant responses to below- and aboveground stresses.

Fig. S11 Power of MTMM in simulations.

Fig. S12 Comparison of SNPs identified by MTMM and Univariate GWAS.

Table S1 Data overview on phenotyping the 350 *Arabidopsis thaliana* accessions of the HapMap collection

Table S2 Summed effect sizes of 30 most significant SNPs in MTMM per trait

Table S3 125 candidate genes derived from the Multitrait Mixed Model analysis

Table S4 Genes in linkage with SNPs with $-\log_{10}$ (P) score above 4 (20 kb half-window size) in the contrast-specific GWA mapping of parasitic plants and aphids on the one hand vs fungus, caterpillar, thrips and drought on the other hand

Table S5 Candidate genes in linkage with SNPs with $–\log_{10}(P)$ score above 4 (20 kb half-window size) that have common effects on plant response to parasitic plants and aphids on the one hand vs fungus, caterpillar, thrips and drought on the other hand

Table S6 Candidate genes in linkage with SNPs with $–\log_{10}(P)$ score above 4 (20 kb half-window size) that have common effects on biotic and abiotic stress responses

Methods S1 Salt.

Methods S2 Abiotic.

Methods S3 Caterpillar – combinatory stress.

Methods S4 Parasitic plants.

Methods S5 Nematodes.

Methods S6 Whiteflies.

Methods S7 Aphids.

Methods S8 Thrips.

Methods S9 Drought – combinatory stress.

Methods S10 Fungus – combinatory stress.

Methods S11 Screening of T-DNA lines.

Methods S12 Simulations to compare power for full MTMM, contrast MTMM and univariate analysis.

Phenotyping the HapMap population of 350 **Arabidopsis thaliana** *accessions*

Methods S1 Salt.

Traits

Salt_1: root response to mild salt stress (75 mM NaCl), in terms of a combination of main root vector length (MRVL), number of lateral roots per main root (noLR) and straightness (main root length divided by MRVL)

Salt_2: root response to severe salt stress (125 mM NaCl), in terms of MRVL

Salt_3: root response to severe salt stress (125 mM NaCl), in terms of noLR

Salt_4: root response to severe salt stress (125 mM NaCl), in terms of straightness.

Methods Table M1 Salt trait reduction overview

Original traits[1]	Variance explained	Trait
Main root vector length at 75 mM NaCl<br>Number of lateral roots per main root at 75 mM NaCl<br>Straightness at 75 mM NaCl	0.585977	Salt_1
Main root vector length 125 mM NaCl	1	Salt_2
Number of lateral roots per main root at 125 mM NaCl	1	Salt_3
Straightness at 125	1	Salt_4

[1]Residuals from control are taken for all original traits.

Growing conditions

Seeds were stratified at 4°C for 3 d. Seeds germinated on square agar plates positioned under an angle of 70 degrees containing half strength Murashi-Skoog medium (MS), 1% Dashin agar, 0.5% sucrose, 0.1% M.E.S. buffer, pH5.8 (KOH). Four-day-old seedlings were transferred into agar plates containing different salt concentrations (0, 75 and 125 mM). After transfer, plants were grown for 8 d at the same conditions as those to which they were exposed when they were germinated, and scanned every second day. The root system architecture was determined of 8-d-old plants in control conditions and 12-d-old plants in both salt stress conditions.

Four-day-old seedlings were transferred to plates containing 0, 75 or 125 mM NaCl (control condition, mild stress, severe stress, respectively). Phenotypes were measured on 8-d-old plants in control conditions and 12-d-old plants in both salt stress conditions. Of each plant root system architecture was determined using EZ-Rhizo software (Armengaud *et al*., 2009).

Experimental design

Plants were screened in seven rounds (experiments), each containing a maximum of 106 accessions. Most of the accessions (198) were present in only one round; Col-0 was present in all rounds. In each round, at least four plants were included per accession-treatment combination. All three treatments (0, 75 or 125 mM NaCl) were screened simultaneously. Plants were allocated to plates, each plate containing two plants of two accessions. The within-plate average of each accession was the basis for subsequent analysis. The position of every plate in the racks was recorded. The growth chamber contained six racks, each holding 64 plates. Positions of racks were also recorded.

Genotypic means

For each of the traits genotypic means were calculated. We obtained BLUEs (best linear unbiased estimator) for all genotype-treatment combinations using the following model:

$$Y = \mu + EXP + TRT + GEN + GEN{:}TRT + EXP{:}TRT + EXP{:}RCK + EXP{:}DIST + GEN{:}EXP + EXP{:}RCK{:}PLT + GEN{:}EXP{:}TRT + E,$$

where *EXP* is experiment, *TRT* is treatment, *GEN* is genotype, *RCK* is rack, *PLT* is plate and *DIST* is distance to the wall. The terms *EXP, TRT, GEN, GEN:TRT* and *EXP:TRT* were modeled as fixed effects and all other terms as random.

Definition of target traits

Stress response was defined as the residual obtained from the regression of the genotypic means for salt stress (either mild or severe) on the values for control conditions. Salt_1 was defined as the first principal component of the response to mild stress of MRVL, noLR and straightness. Salt_2, Salt_3 and Salt_4 were defined as the severe stress of MRVL, noLR and straightness individually.

Methods S2 Abiotic.

Traits

Salt_5: plant response to mild salt stress (25 mM NaCl), in terms of plant fresh weight, dry weight and water content

Drought_1: plant response to drought stress (0.22 g H_2O g^{-1} soil at day 14), in terms of plant fresh weight and water content

Osmotic: plant response to osmotic stress (10% of PEG8000 from day 8 until 18), in terms of fresh weight, dry weight, water content and rosette area

Heat: plant response to heat stress (1 d, 35°C), in terms of number and length of siliques

Methods Table M2 Abiotic trait reduction overview

**Original traits**	**Variance explained**	**Trait**
Fresh Weight of the Rosette at day 28	0.789968	Salt_5
Dry Weight of Rosette at day 28		
Water Content of Rosette at day 28		
Dry weight of largest leaf at day 24		
Fresh Weight of Rosette at day 28	0.541105	Drought_1
Fresh Weight of largest leaf at day 24		
Rehydrated Weight of largest leaf at day 24		
Water Content of the largest leaf at day 24		
Dry Weight of Rosette at day 28[2]	0.679514	Osmotic
Fresh Weight of Rosette at day 28[2]		
Water Content of Rosette at day 28[2]		
Rosette Area at day 28[2]		
Rosette Area at day 28 without bolting plants.[2]		
Number of aborted siliques along the inflorescence	0.647069	Heat
Number of silique (<5mm) along the inflorescence		
Number of silique (<5mm) in the region -10 until 20. [1]		
Average of the length of all siliques along the inflorescence		
Average of the length of siliques 0 until 10[1]		
Average of the length of siliques 0 until 20[1]		
Average of the length of siliques 10 until 20[1]		
Average of the length of siliques 20 until 30[1]		
Average of the length of siliques -10 until 0[1]		

[1]Silique zero belongs to the flower that opened first on the day of the treatment.

[2]Stress did not disappear when watering with PEG-containing nutrient solution stopped, because PEG is not evaporating.

Growing conditions

Four types of stress were studied in four different experiments. Seeds were sown in Petri dishes on wet filter paper. After 4 d of cold treatment, they were placed in the light at room temperature for 1.5 d to germinate. Germinated seeds were placed on rockwool blocks saturated with nutrient solution (Hyponex, 1mM N, 1.1 mM P, 5.9 mM K). For the stress treatments control plants received nutrient solution only. Salt treatment contained Hyponex + 25 mM NaCl. The plants of the salt experiments were automatically watered by a flooding system three times a week. For the osmotic treatment, the plants received nutrient solution containing 0.1 g ml^{-1} PEG8000 on day 8, 11, 13 and 15. For the other experiments plants automatically watered by a flooding system for *c*. 5 min, three times a week. All experiments were performed under 125 µmol m^{-2} s^{-1} light, 16 h 20°C: 8 h 18°C, light : dark schedule, and 70% humidity.

Experimental design

In the salt experiment three blocks received control treatment and three blocks received treatment conditions. For the drought experiments the Phenopsis phenotyping platform was used, preventing position related differences in plant growth within the climate chamber (Granier *et al*., 2006; Bac-Molenaar *et al*., 2015). The plants were grown in four rounds of 84 accessions. Four of these 84 were used for reference accessions, which were grown in each round. Each round contained three blocks and all 84 accessions were present in each block. Plants for the PEG experiment were grown in six blocks. Each accession was present in each block. Within the block the plants were grown in nine trays each containing 40 plants. Within a tray the plants had a fixed position. The nine trays were positioned randomly within the block. Three blocks received PEG treatment and three blocks received control treatment.

For the heat experiment, eight plants of each accession were grown in controlled conditions (Bac-Molenaar *et al*., 2015). Five replicates received a heat treatment and three replicates served as controls. One to 2 wk after the first replicate of each accession started to flower, the heat treatment was applied. A small number of accessions received the treatment outside this window. The first flower that opened first on the day of the treatment was tagged with a thread. Three replicates per accession were kept in the climate room as controls. Five replicates per accession were transferred to a climate cabinet where they received heat treatment. At the start of the day, the temperature was raised from 20°C to 35°C within 2 h. The temperature was kept at 35°C for 13.5 h. At the end of the light period, the temperature was decreased again to 20°C in 2 h. The day after the treatment the plants returned to the climate room.

Genotypic means and definition of target traits

All data in Drought_1 are log-transformed. In the Salt and PEG experiments, genotypic means were calculated using a mixed model containing random block effects and genotypic fixed effects. For the Heat experiment, the model included fixed effects for treatment, genotype and genotype x treatment interaction. For the Drought experiment, we fitted the mixed model used in (Bac-Molenaar *et al*., 2015). Next, for all traits in the four experiments, the impact of the stress was quantified using the residuals from the regression of genotypic means under stress on those under control conditions, except for the heat traits 'Rosette Area at day 28',

where no control was available. Finally, the four target traits were defined as the first principal component of all traits (residuals) from the corresponding experiments.

Methods S3 Caterpillar – combinatory stress.

Traits

Drought_2:	Plant response to drought (7 d), in terms of plant biomass after drought recovery
Caterpillar_2:	Plant response to *P. rapae*, in terms of plant biomass
Fungus and caterpillar_1:	Plant response to *Botrytis cinerea* and *P. rapae*, in terms of plant biomass
Caterpillar_3:	Plant response to *P. rapae*, in terms of damaged leaves and feeding sites
Drought and caterpillar:	Plant response to drought stress and *P. rapae*, in terms of damaged leaves and feeding sites
Fungus and caterpillar_2:	Plant response to *B. cinerea* and *P. rapae*, in terms of damaged leaves and feeding sites

Methods Table M3 Caterpillar – combinatory stress trait reduction overview

**Original traits**	**Variance explained**	**Trait**
Biomass reduction (with respect to control conditions) upon drought stress following a recovery period.	1	Drought2
Biomass reduction (with respect to control conditions) upon *P. rapae* herbivory	1	Caterpillar_2
Biomass reduction (with respect to control conditions) upon *P. rapae* herbivory preceded by *B.cinerea*<br>Biomass reduction (with respect to *P. rapae* single stress) upon *P. rapae* herbivory preceded by *B.cinerea*	0.910749	Fungus & caterpillar_1
Number of leaves damaged upon *P. rapae* herbivory<br>Number of feeding sites upon *P. rapae* herbivory	0.792034	Caterpillar_3
Number of leaves damaged upon *P. rapae* herbivory preceded by drought<br>Number of feeding sites upon *P. rapae* herbivory preceded by drought	0.792354	Drought & caterpillar
Number of leaves damaged upon *P. rapae* herbivory preceded by B.cinerea<br>Number of feeding sites upon *P. rapae* herbivory preceded by *B.cinerea*	0.788294	Fungus & caterpillar_2

Growing conditions

Plants were grown for 4 wk at 23°C, 70% RH, 100 μmol m^{-2} s^{-1} light intensity and 8h:16h L:D photoperiod.

Plants were grown under similar conditions during the first 3 wk. Drought stress was imposed by withholding water for 7 d while the rest of plants were watered every 2 d with 1 l of water per tray. *Botrytis* inoculation was carried out 24 h before *Pieris* inoculation. Plants were 4 wk old when they were exposed to stress by *P. rapae* as single or combined stress. Plants were inoculated with two newly hatched L1 and the larvae were allowed to

feed for 5 d until harvesting. Phenotypic measurements were taken 24 h and 5 d after inoculation with *P. rapae* as a single and combined stress. After 24 h, the number of leaves damaged and number of feeding sites was counted in plants exposed to *P. rapae*, drought and *P. rapae,* and *Botrytis* and *P. rapae*. After 5 d, fresh weight was measured for the five treatments.

Experimental design

Plants were screened in rounds of 37 accessions. Three control accessions were present in all rounds (Col-0, Tsu-0, Fei-0). In each round, six replicates were included per accession-treatment combination. Treatments were screened simultaneously. Plants were randomly allocated in trays (28 accessions per tray). Plant positions within a tray were recorded (X_{pos} and Y_{pos}). The chamber where the experiment were conducted consist of six racks**,** and each rack contained four shelves**.** Positions of trays within shelves within racks were also recorded.

Genotypic means

For each of the three traits (shoot fresh weight, number of leaves damaged and number of feeding sites) we fitted the following mixed model:

$$Y = \mu + ROUND + RACK + SHELF + TRT + GEN + GEN{:}TRT \\ + ROUND{:}RACK{:}SHELF + ROUND{:}RACK{:}SHELF{:}TRAY + ROUND{:}RACK{:}SHELF{:}TRAY{:}X_{POS} \\ + ROUND{:}RACK{:}SHELF{:}TRAY{:}Y_{POS} + E,$$

where *TRT* is the treatment factor (Control and four treatment levels), *GEN* is genotype (accession) and *GEN:TRT* is the genotype by environment interaction. The terms *GEN, TRT* and *GEN:TRT* were fitted as a fixed effect and all others as random. For each of the three traits, significance of each model term was assessed and only significant terms were retained for the estimations of genotypic means. Genotypic means (BLUEs) were calculated for shoot fresh weight, number of leaves damaged and number of feeding sites, for each accession-treatment combination.

Definition of target traits

Target traits were defined based on the genotypic means for shoot fresh weight, number of leaves damaged and number of feeding sites. The traits Caterpillar_3, Drought & caterpillar and Fungus & caterpillar_2 were defined as the first principal component of the number of damaged leaves and number of feeding sites under the respective types of stress. For shoot fresh weight, stress response was defined by the residuals obtained from the regression of genotypic means under each stress condition on those for the non-stress condition (Drought2, Caterpillar_2 and Fungus & caterpillar_1). In case of Fungus & caterpillar_1, also the regression of the double stress (*P. rapae* and *B. cinerea)* on the single stress (*P. rapae)* was performed, and the trait was defined as the first principal component of the two residuals.

Methods S4 Parasitic plants**.**

Trait

Parasitic plant: Plant response to parasitic plant (*Phellipanche ramose*), in terms of the total number of parasitic plant organ attachments onto the host root

Growing conditions

Arabidopsis seeds were put on filter paper in the dark at 4°C for 2 d. Then, Arabidopsis seeds were sown on river sand (with a thin layer of soil on the top of river sand). Arabidopsis plants were grown for 2 wk on river sand at 21°C, 60% RH, 100 µmol m^{-2} s^{-1} light intensity, 12 h : 12 h, light : dark photoperiod. After 2 wk, Arabidopsis seedlings were surface-sterilized with 70% ethanol for 5 s and washed with sterile demi-water. The rhizotron system was prepared by cutting a hole at the side of 14.5 cm diameter round Petri dish, putting successively a piece of round rock wool slice (14.5 cm diameter, 1.5 cm in thickness) at the bottom of Petri dish, a piece of 12 cm diameter glass-fibre filter discs and a piece of 14.5 cm diameter nylon mesh on top. The rhizotron system was supplied with sterile ½ Hoagland liquid medium. Sterile seedlings were then moved to prepared rhizotron system by fitting the plant in the hole of the Petri dish. Leaves and shoots of the seedlings were kept outside of Petri dishes. The roots were carefully separated and organized on the top of nylon mesh by forceps. Arabidopsis seedling were grown in rhizotron system at 21°C, 60% RH, 100 *µ*mol m^{-2} s^{-1} light intensity, 12 h : 12 h, light : dark photoperiod for another 2 wk.

Sterile *Phellipanche ramosa* seeds were spread on 5 cm diameter glass-fiber filter discs (Whatman GF/A paper) which were wetted with 0.8 ml sterilized demi-water and placed in 9 cm diameter Petri dishes. The Petri dishes were sealed with parafilm and then kept in dark in a growth chamber at 20°C for a 12 d precondition period. Preconditioned seeds on a glass-fiber filter disc were dried and treated with 0.8 ml strigolactone analog GR24 at the concentration of 3.3 x 10^{-3}µM for 1 d under dark at 25°C. GR24 treatment triggered the initial germination of *P. ramosa*. After 1 d, GR24 was immediately washed off the *P. ramosa* seeds by sterile demi-water.

Pre-germinated *P. ramosa* seeds were spread along 4-wk-old Arabidopsis seedlings in the rhizotron system with painting brushes. The rhizotron Petri dish were sealed with tape and covered by aluminium foil. Plant were grown at the same condition for the following 4 wk. Pictures of *P. ramosa*-infested roots in the rhizotron system were taken 4 wk after infection with Canon camera EOS 60D DSLR (with EF-S 18-135 mm IS Lens).

Experimental design

The 359 accessions were screened in two rounds (the first 200 accessions, the second with 160 accessions, two accessions were used for control in both rounds). Rhizotron Petri dishes were randomly arranged in trays. Positions of trays and Petri dishes were rearranged randomly every 3 d. Pictures of rhizotrons were taken after 4 wk. Image analysis was done with the ImageJ software (Schneider *et al*., 2012). The number of attachment organs was counted. The total number of pre-germinated *P.ramosa* seeds was recorded as a co-variable.

Genotypic means and definition of target traits

Values for diameter of attachment organs, number of attachment organs and number of pre-germinated seeds were log-transformed for normality and were averaged over technical replicates where present. Since there was significant correlation between the two variables of interest and the number of pre-germinated *P.ramosa* seeds, we used the residuals from the regression on the number of pre-germinated seeds for further analysis.

Methods S5 Nematodes.

Trait

Nematode: Plant response to nematodes (*Melodoigyne incognita*, 180 stage-2 juveniles), in terms of number of *M. incognita* egg masses per plant.

Growing conditions

Seeds were vapor-sterilized for 5 h and transferred to a six-well plate with MS20 (5% gelrite). After four nights in the dark at 4°C the plates were transferred to 24°C in 12 h light. At the age of 1 wk the seedlings were transferred individually to a well of a six-well plate. *Melodoigyne incognita* infection was induced with 180 juveniles stage-2 added to 2-wk-old seedlings. Six-well plates with nematodes and seedlings were incubated in the dark at 24°C for 6 wk. Plants were grown for 2 wk 24°C, 12 h : 12 h, light : dark, then 6 wk 24°C, dark. Egg masses were quantified manually.

Experimental design

Plants were screened in rounds of 20 accessions. Each round included a six-well plate with one Col-0 plant as reference.

Genotypic means

'Nematode' was defined as the number of egg masses, after arcsine-square root transformation. Genotypic means were calculated using the following mixed model:

$Y = \mu + GEN + RND + E,$

where GEN is genotype (accession) and *RND* is a random effect for round.

Methods S6 Whiteflies**.**

Traits

Whitefly_1: Plant response to whitefly (*Aleyrodes proletella*, five females), in terms of whitefly survival

Whitefly_2 Plant response to whitefly (*Aleyrodes proletella*, five females), in terms of number of eggs

Growing conditions

Plants were grown for 5 wk at 20°C, 70% RH, 100 µmol m^{-2} s^{-1} light intensity and 10 h : 14 h, light : dark photoperiod. One leaf of each accession was infested with five female whiteflies (placed in clip cages) that were allowed to feed and oviposit. Seven days after infestation, the number of living and dead females was counted as well as the number of eggs. From this, we calculated the survival (number of living flies divided by the total number of flies) and oviposition rate (eggs laid per female d^{-1}).

Experimental design

Accessions were screened in three blocks of 120 accessions with five reference accessions (Col-0, Ler-1, WS-0, Cvi-0, Kin-0) in each block. The whole experiment was repeated five times.

Genotypic means

Genotypic means were calculated with Genstat 15th edition (Payne, 2009), using the following mixed model:

$Y = \mu + REP + GEN + REP{:}BLOCK + E$,

where *GEN* is genotype (accession), *REP* denotes complete replicates (experiments with three blocks) and *REP:BLOCK is a random effect for incomplete blocks within replicates.*

Methods S7 Aphids.

Traits

Aphid_1 Plant response to aphids (*Myzus persicae*), in terms of behavior at t1

Aphid_2 Plant response to aphids (*M. persicae*), in terms of behavior at t2

Aphid_3 Plant response to aphids (*M. persicae*), in terms of aphid reproduction

Methods Table M4 Aphid trait reduction overview

Original traits	Variance explained	Trait
Total duration probing (logit) [1]<br>Total duration of short probes (< 3 min , arcsine) [1]<br>Total duration of intermediate probes (< 15 min, arcsine) [1]	0.74997	Aphid_1
Total duration probing (logit) [2]<br>% of aphids making long probes (>= 15 min) [2]<br>Total duration of intermediate probes (< 15 min, arcsine)[2]	0.655366	Aphid_2
Number of aphids per plant [3]	1	Aphid_3

[1]0 h after inoculation.

[2]4.5h after inoculation.

[3]2 wk after inoculation.

Growing conditions

Plants were grown for 4–5 wk at 23°C, 70% RH, 200 µmol m^{-2} s^{-1} light intensity and 8 h : 16 h, light : dark photoperiod. Green peach aphids, *M. persicae*, were reared on radish, *Raphanus* sativus, at 19°C, 50–70% relative humidity and a 16 h : 8 h, day : night cycle. Behaviour of the green peach aphid was screened by automated video-tracking. One leaf disc was collected per plant from an intermediately aged leaf and placed abaxial side up on a 1% agar substrate in a well of a 96-well microtitre plate. *Myzus persicae* was reared on radish, *Raphanus sativus*, at 19°C, 50–70% relative humidity and a 16 h : 8 h, day : night cycle. One 7- to 8-d-old wingless aphid was released on the leaf disc and cling film was used to cover the arena. Twenty Arenas were recorded simultaneously with a mounted camera. Aphids were observed for 85 min on two time points: (1) immediately after introducing the aphids into the arenas; and (2) 4.5 h after the start of the first observation. Motion analysis was performed with EthoVision XT® 8.5 software (Noldus Information Technology bv, Wageningen, The Netherlands). Aphids are phloem-feeding insects and probe with their piercing mouthparts between plant cells to feed from the plant sap. Start time and duration of probes were registered with automated video-tracking (Kloth *et al.*, 2015). Aphid survival was checked 24 h after recording. Subject detection was checked on four time points within each movie. Samples with no survival, low subject detection or with less than five replicates were excluded from analysis. Probes were categorized into short (<3 min) probes and intermediate (<15 min) probes, both associated with penetration of the plant epidermis or

mesophyll, and long (≥15 min) probes, putatively associated with phloem uptake. Response variables expressed in seconds were arcsin or logit transformed to approach a normal distribution.

Aphid reproduction was measured in a whole-plant assay. Each 2-to-3-wk-old plant was inoculated with one 0-to-24-h-old nymph. Two weeks after infestation, aphid population size was measured per plant. Plants were placed in a Petri dish in trays with a water barrier to prevent aphids to move between plants. Each tray contained 20 plants, none of the aphids developed wings.

Experimental design

Automated video tracking of aphid behaviour was performed in an incomplete block design with each complete replicate consisting of 18 incomplete blocks of 20 accessions. One replicate of the complete Hapmap collection was acquired in 6 d, 60 plants were screened each day across three batches. An alpha design was generated with Gendex (http://designcomputing.net/gendex/) to assign accessions to blocks. For each accession five to six replicates were acquired.

Phenotyping of aphid reproduction was performed in an incomplete block design with seven incomplete blocks. Blocks were defined according to the position in the climate cell. Each replicate consisted of three to four blocks and plant genotypes were randomized across blocks between replicates. For each genotype two to three replicates were acquired.

Genotypic means and definition of target traits

Genotypic means were calculated using the following linear mixed model:

$$Y = \mu + REP + GEN + REP{:}BLOCK + E,$$

where REP denotes complete replicate and REP:BLOCK is a random term for block nested within replicate.

Methods S8 Thrips.

Traits

Thrips_1	Plant response to thrips (*Frankliniella occidentalis*, three juveniles, 6 d), in terms of feeding damage on detached leaf.
Thrips_2	Plant response to thrips (*F. occidentalis* one adult), in terms of behavior/ preference at t1 (0 hpi) in two choice leaf disc assay
Thrips_3	Plant response to thrips (*F. occidentalis*, one adult), in terms of behavior/ preference at t2 (4 hpi) in two choice leaf disc assay

Growing conditions

Plants were grown for 5 wk at 23°C, 70% RH, 200 µmol m^{-2} s^{-1} light intensity and a 8 h : 16 h, light : dark photoperiod.

For trait 'Thrips_1', feeding damage on detached leaves was scored. Leaves were cut from plants, and kept turgid in Petri dishes with a diameter of 5 cm (BD falcon, Product Number: 351006) containing a film of 1% technical agar. The amount of feeding damage on one leaf was manually scored after 6 d of exposure to three juvenile thrips.

For traits 'Thrips_2' and 'Thrips_3', thrips preference was phenotyped with an automated video tracking setup. Thrips behavior was tracked in two-choice arenas using 96-well plates, consisting of halved leaf discs from Col-0 and one of the HapMap accessions. Position bias was corrected for, by alternating the Col-0 leaf disc position (left or right) every row. 20 plants were screened in one recording. Thrips position was automatically monitored for 40 min (Thrips_2), and once more for 40 min after 4 h (Thrips_3). The ratio of time spent on Col-0 was used for Thrips_2 and Thrips_3. Video tracking was performed with EthoVision XT 8.5 software (Noldus Information Technology bv, Wageningen, The Netherlands).

Experimental design

Plants were screened in five rounds (complete replicates) of 360 accessions, using an incomplete block (alpha) design. Within each round plants were randomly allocated to 18 blocks of 20 accessions, the blocks representing plants being screened in one recording. One sampling day consisted of five blocks (100 accessions), with the exception of the last day (three blocks, 60 accessions).

Genotypic means

Genotypic means were calculated using the following linear mixed model:

$$Y = \mu + REP + GEN + REP{:}BLOCK + E,$$

where REP denotes complete replicate and REP:BLOCK is a random term for blocks nested within replicate.

Methods S9 Drought – combinatory stress.

Traits

Caterpillar_1:	Plant response to *Pieris rapae* change in terms of rosette area
Caterpillar and osmotic_1:	Plant response to *P. rapae* and osmotic stress (PEG8000), in terms of terms of rosette area
Caterpillar and osmotic_2:	Plant response to *P. rapae* and osmotic stress (PEG8000), in terms of plant biomass

Methods Table M5 Drought – combinatory stress trait reduction overview

**Original traits**[1]	**Variance explained**	**Trait**
Rosette perimeter after Caterpillar treatment<br>Rosette area after Caterpillar treatment<br>Rosette ferret after Caterpillar treatment	0.841488	Caterpillar_1
Rosette perimeter after Caterpillar/Osmotic treatment<br>Rosette area after Caterpillar/Osmotic treatment<br>Rosette ferret after Caterpillar/Osmotic treatment	0.823736	Caterpillar & osmotic_1
Plant Fresh weight after Caterpillar/Osmotic treatment	1	Caterpillar & osmotic_2

[1]Residuals obtained from regressing treatment means on control means.

Growing conditions

Plants were grown for 4 wk at 21°C (day temperature) and 19°C (night temperature), 70% RH, 200 µmol m^{-2} s^{-1} light intensity, and 10 h : 14 h SD photoperiod. Projected leaf area, rosette feret and rosette perimeter were measured using ImageJ software in the *P. rapae* and PEG8000 combined treatment group (Schneider *et al*., 2012). Data were recorded at three time points: T1, before applying *P. rapae*; T2, before applying PEG8000 treatment; T3, after 7 d PEG8000 treatment. Rosette fresh weight was measured from both control and combinatorial stress treatment groups at T3. We used 332 Arabidopsis accessions, grown on rock wool blocks in a climate controlled growth chamber. Plants were first treated with *P. rapae* L1 larvae for 24 h, and then irrigated with nutrient solution that containing PEG8000 for 7 d (*P. rapae* and PEG8000 combined stress treatment). In additional, plants were grown without any stress treatment (control).

Experimental design

All traits were measured in a randomized complete block design with two complete blocks (replicates) under treatment conditions and 2 complete blocks under control conditions.

Genotypic means

The square root transformation was first applied to the area traits. For each treatment, genotypic means were calculated, using a linear model with a fixed effect for block.

Definition of target traits

We regressed the genotypic means of fresh weight at T3 after *P. rapae* and PEG8000 treatment on the means of fresh weight at T3 under control conditions; these residuals represent the effect of *P. rapae* and PEG8000 treatment on fresh weight (Caterpillar_&_osmotic_2). Similarly, we regressed each of the rosette area related traits (projected leaf area, rosette feret, and rosette perimeter) observed at T2 (only *P. rapae* treatment) on the corresponding means of these traits measured at T1 (Caterpillar_1). The resulting residuals represent the effect of *P. rapae* treatment on rosette area related traits. Finally we performed the regression of rosette area related traits at T3 on the values measured at T1 as well as T2, whose residuals represent the combined effect of *P. rapae* and PEG8000 treatment on rosette area related traits (Caterpillar_&_osmotic_1). In both the Caterpillar_1 and Caterpillar_&_osmotic_1 group, the three traits were replaced by the first principal component.

Methods S10 Fungus – combinatory stress.

Traits

Fungus: Plant response to *B. cinerea* infection (1 x 10^5 spores ml^{-1}), in terms of percentage of spreading lesions

Drought and fungus: Plant response to drought (7 d) followed by *B. cinerea* infection, in terms of percentage of spreading lesions

Caterpillar and fungus: Plant response to *P. rapae* feeding (one L1 caterpillar per plant, 24 h) followed by *B. cinerea* infection, in terms of percentage of spreading lesions

Growing conditions

Seeds were sown and vernalized for two d at 4˚C on river sand supplied with half strength Hoagland medium with sequestreen. Ten-day-old seedlings were transplanted to pots containing half volume river sand and half volume sowing soil supplemented with Hoagland solution (with sequestreen). Plants were kept at *c*. 21˚C, 70% relative humidity, 8 h : 16 h, light : dark period. At day 0 of the experiment 27-d-old plants were exposed to a period of drought stress or a normal watering regime. At day 7 of the experiment, drought stress was stopped by re-watering the drought stressed plants. At that day 7 one first instar (L1) *P. rapae* caterpillar was put on each plant for the dual stress combination with herbivory. At day 8, *P. rapae* was taken off the plants and all the plants from the different treatments were simultaneously inoculated with *B. cinerea*. Six leaves per plant were each drop inoculated with 5 µl of 1 × 10^5 spores ml^{-1}, in half strength potato dextrose broth. Plants were kept under *c*. 100% humidity for 3 d, after which the disease severity was measured on day 11 of the experiment. Severity was measured as percentage of leaves with spreading lesions caused by *B. cinerea*. In total six leaves per plant were scored. Lesions that did not exceed the size of the droplet, (5 µl) were scored as zero, whereas a spreading lesion was scored as a one.

Experimental design

Plants were screened in rounds of 35 accessions. Col-0 was present in all rounds as a control. The three treatments were screened simultaneously.

Genotypic means and definition of target traits

An arcsine transformation was applied to the proportion of leaves with spreading lesions, that is, for each observed count k = 0,1,2,3,4,5,6, the transformed phenotype was defined as $\arcsin\sqrt{((k)/6)}$. Before transformation, counts equal to zero or 6 were replaced by respectively 1/4 and 5.75=6-1/4. The transformed phenotypic observations were corrected for round effects by subtracting from each observation the mean of the round it was contained in, and genotypic means were calculated based on the round corrected phenotypes. Differential sensitivity of each double stress was calculated as the residuals obtained from the linear regression of the double stress on the single stress phenotype.

Methods S11 Screening of T-DNA lines.

T-DNA lines were ordered and screened for homozygosity, using primers described in Methods Table M6. Seeds from homozygous mutants were harvested and grown and screened individually by consortium partners (Methods Table M7). The top 300 coexpressed genes of RMG1 were retrieved from Atted-II version 8.0 (Obayashi *et al*., 2014). GO enrichment analysis was performed with the application BiNGO in Cytoscape (Cline *et al*., 2007; Maere *et al*., 2005).

Methods Table M6 T-DNA lines and primers

**Gene**	**N number**	**mutant line**	**LP**	**TM**	**RP**	**TM**	**product size**
WRKY38	N864818	WiscDsLox489-492C21	ATTTGGTAAACCCAAATTGGC	59.94	CGATGAAGGAGGATAAGAGCC	60.18	1178
TOUCH4	N860818	SAIL_158_A07	AACAAAAACCGCGTGATTTC	59.98	CAAGAAGACTTGCCGTTTGAC	59.91	1010
TOUCH4	N860819	SAIL_422_D11	AACAAAAACCGCGTGATTTC	59.98	CAAGAAGACTTGCCGTTTGAC	59.91	1010
RMG1	N674117	SALK_023944.54.15.x	TGGTCTAATGGGCTCAATGAG	60.08	CATAGCCGTTGTCAATTCCAG	60.51	1009
RMG1	N678063	SALK_007034.41.00.x	TTTAGCGGTCAACACGAAAAC	60.16	CCAAAATTGAAAATAGAGAACCC	58.14	1196

Methods Table M7 Methodology screening of T-DNA mutants

**Trait**	**Number of replicates**	**Method**
Thrips	24	See section 3.7, Thrips_1
Aphids	10-17	See section 3.6, Aphid_3
Whitefly	10	See section 3.5, Whitefly_1
Caterpillar	6	For the caterpillar treatment, each plant was exposed to 1*Pieris rapae* 1st instar larvae for 24h, thereafter, the caterpillar was removed from the plant. Damage was assessed using ImagJ software.
Nematodes	23	See section 3.3, Nematode
Salt	10	See section 3.2, Salt_5. 75 mM Salt instead
Drought	4	Plants were irrigated with Hyponex solution containing 7.7% polyethylene glycol (PEG8000) of osmotic potential *c*. 0.1MPa for 7 d.

Methods S12 Simulations to compare power for full MTMM, contrast MTMM and univariate analysis.

To compare the performance of the full and contrast MTMM, we repeatedly simulated 30 traits for all of the *n* = 350 Hapmap accessions, for two scenarios. In scenario A, SNP-effects had the same sign within two pre-defined groups of 15 traits, whereas in scenario B, each SNP-effect was given a randomly chosen sign. For both scenarios, each of the 1000 simulations was performed as follows:

- The 30 x 30 matrix V_g containing the genetic covariances was simulated using a first order factor analytic model: $V_g = \lambda\lambda^t + diag(\tau_1^2, \dots, \tau_p^2)$, where both the elements of λ and the diagonal elements τ_j where drawn independently from the standard normal distribution. Next, we have randomly drawn the heritabilities of the 30 traits from the uniform distribution on the interval [0.2, 0.7], and defined the environmental covariance matrix $V_e = diag(\sigma_{e,1}^2, \dots, \sigma_{e,p}^2)$, choosing the diagonal elements in order to achieve the given heritability.
- Given V_g and V_e , we simulated the 350 x 30 matrices G and E, containing respectively the genetic and residual effects. G followed a matrix normal distribution with row- and column covariance K and V_g , where K is the 350 x 350 genetic relatedness matrix. Equivalently, the length 10500 vector $vec(G)$ obtained by stacking all columns of G followed a multivariate normal distribution with covariance $V_g \otimes K$. Similarly, $vec(E)$ followed a zero mean normal distribution with covariance $V_e \otimes I_n$.
- We randomly selected one of the 214051 available SNPs, under the restriction that its minor allele frequency was at least 0.4. This restriction is not essential, and only serves to avoid extra variation in the simulation results due to varying allele frequencies (which affect power).
- We defined a 350 x 30 matrix S by multiplying the vector of SNP-scores (x) with trait specific SNP-effects: the *j*th column of S was defined as $x\,\beta_j$. The magnitude of the effects β_j was chosen such that the explained variance for the *j*th trait was 1% of the polygenic variance of that trait (i.e. the *j*th diagonal element of V_g). In scenario A, the effects were made negative for the first 15 traits and positive for traits 16,...,30, whereas in scenario B each SNP-effect was given a randomly chosen sign.
- The matrix of phenotypes was defined as $Y = G + E + S$, which was used for all subsequent analyses.
- We fitted a first-order analytic model without marker effects, using the 37 x 37 compressed kinship matrix used for the full MTMM in the main text (for computational reasons the latter was kept constant throughout all simulations).
- As in the MTMM analyses in the main text, we tested the significance of marker effects conditional on the variance components estimated in the previous step. Using the Wald test, we tested the hypothesis $\beta_1 = \beta_2 = \cdots \beta_p = 0$ ('full MTMM') as well as the hypothesis $\alpha_{group\,1} = \alpha_{group\,2}$ in the restricted model where marker effects within groups are equal ('contrast MTMM'). For comparison, we also performed a univariate Wald test on the trait with the highest heritability, where we estimated the polygenic variance component using the complete (uncompressed) 350 x 350 relatedness matrix (i.e. similar to emma-x (Kang *et al*. 2010) or Fast-LMM (Lippert *et al*. 2012)).

In both scenarios, the power of the full MTMM, contrast MTMM and univariate analysis was estimated by the proportion of simulations where the $-\log_{10}(P)$ value was above a certain threshold.

Table S1 Data overview on phenotyping the 350 *Arabidopsis thaliana* accessions of the HapMap collection

Trait	Section[(1)]	Number of accessions[(2)]	H^2 [(3)]	h^2 [(4)]	L 95% CI h^2	R 95% CI h^2
Salt_1	2.1	328	NA	0.60	0.22	0.89
Salt_2	2.1	323	0.78	0.43	0.15	0.77
Salt_3	2.1	323	0.68	0.64	0.27	0.89
Salt_4	2.1	322	0.43	0.30	0.08	0.68
Fungus	2.10	336	0.79	0.40	0.13	0.74
Drought & fungus	2.10	336	NA	0.31	0.08	0.68
Caterpillar & fungus	2.10	336	NA	0.17	0.03	0.55
Heat	2.2	275	NA	0.62	0.25	0.89
Osmotic	2.2	346	NA	0.10	0.004	0.75
Drought_1	2.2	323	NA	0.39	0.12	0.75
Salt_5	2.2	334	NA	0.15	0.01	0.76
Whitefly_1	2.5	339	0.85	0.01	0.00	1.00
Whitefly_2	2.5	339	0.87	0.01	0.00	1.00
Aphid_1	2.6	341	NA	0.10	0.004	0.76
Aphid_2	2.6	341	NA	0.36	0.08	0.79
Aphid_3	2.6	337	0.48	0.19	0.03	0.66
Thrips_1	2.7	346	0.44	0.80	0.37	0.96
Thrips_2	2.7	347	0.00	0.14	0.01	0.66
Thrips_3	2.7	346	0.06	0.29	0.06	0.73
Caterpillar_1	2.8	328	NA	0.15	0.01	0.78
Caterpillar &osmotic_1	2.8	326	NA	0.08	0.003	0.72
Caterpillar &osmotic_2	2.8	324	NA	0.08	0.002	0.82
Drought_2	2.9	346	NA	0.06	0.002	0.66
Caterpillar_2	2.9	346	NA	0.23	0.04	0.68
Fungus & caterpillar_1	2.9	346	NA	0.20	0.03	0.64
Caterpillar_3	2.9	346	NA	0.27	0.06	0.69
Drought & caterpillar	2.9	346	NA	0.28	0.07	0.67
Fungus & caterpillar_2	2.9	346	NA	0.10	0.005	0.72
Nematode	2.3	313	0.86	0.72	0.35	0.93
Parasitic_plant	2.4	232	NA	0.03	0.00	1.00

[1]Section in Supporting Information methods where additional information on phenotyping can be found.

[2]Number of accessions included in the analyses.

[3]Broad-sense heritability estimates.

[4]Narrow sense heritability estimated using the 'heritability' R package.

Table S2 Summed effect sizes of 30 most significant SNPs in MTMM per trait

Trait	Stress	Summed absolute effect size
Caterpillar_2	Single	3.42
Drought_1	Single	3.59
Caterpillar_1	Single	3.81
Aphid_2	Single	3.99
Salt_1	Single	4.13
Drought_2	Single	4.25
Whitefly_2	Single	4.29
Heat	Single	4.37
Thrips_3	Single	4.42
Whitefly_1	Single	4.51
Aphid_1	Single	4.54
Fungus and Caterpillar_1	Double	4.67
Salt_5	Single	4.99
Nematode	Single	5.09
Parasitic plant	Single	5.11
Salt_2	Single	5.11
Thrips_2	Single	5.19
Fungus and Caterpillar_2	Double	5.21
Osmotic	Single	5.30
Aphid_3	Single	5.33
Caterpillar_3	Single	5.44
Caterpillar and osmotic_2	Double	5.69
Thrips_1	Single	6.03
Salt_4	Single	6.06
Caterpillar and osmotic_1	Double	6.17
Salt_3	Single	6.77
Drought and Caterpillar	Double	7.42
Drought and fungus	Double	10.06
Fungus	Single	10.09
Caterpillar and fungus	Double	11.93

Table S3 One hundred and twenty-five candidate genes derived from the Multitrait Mixed Model analysis; stress-responsive genes are highlighted in yellow

**Significant SNP or gene in LD**	**Associated marker**	**Gene**	**Gene name**	**Gene description**
Significant SNP	Ch1: 25500708	AT1G68030		RING/FYVE/PHD zinc finger superfamily protein
Significant SNP	Ch1: 26798534	AT1G71040	Low Phosphate Root2 (*LPR2*)	Encodes LPR2. Function together with LPR1 (AT1G23010) and a P5-type ATPase (At5g23630/PDR2) in a common pathway that adjusts root meristem activity to inorganic phosphate availability
Significant SNP	Ch1: 29518622	AT1G78460		SOUL heme-binding family protein
Significant SNP	Ch1: 3294935	AT1G10090		Early-responsive to dehydration stress protein (ERD4)
Significant SNP	Ch1: 7207918	AT1G20750		RAD3-like DNA-binding helicase protein
Significant SNP	Ch2: 11531255	AT2G27020	20S proteasome alpha subunit G1 (*PAG1*)	Encodes 20S proteasome alpha 7 subunit PAG1
Significant SNP	Ch2: 11659416	AT2G27240		Aluminium-activated malate transporter family protein
Significant SNP	Ch2: 391904	AT2G01880		Purple acid phosphatase 7 (PAP7)
Significant SNP	Ch3: 1077306	AT3G04110	glutamate receptor 1.1 (*GLR1.1*)	Putative glutamate receptor (GLR1.1). Contains a functional cation - permeable pore domain. Involved in cellular cation homeostasis.
Significant SNP	Ch3: 18615891	AT3G50210		2-oxoglutarate (2OG) and Fe(II)-dependent oxygenase superfamily protein
Significant SNP	Ch3: 19804402	AT3G53420	plasma membrane intrinsic protein 2A (*PIP2A*)	Member of the plasma membrane intrinsic protein subfamily PIP2. Localizes to the plasma membrane and exhibits water transport activity in *Xenopus* oocyte. Expressed specifically in the vascular bundles and protein level increases slightly during leaf development. When expressed in yeast cells can conduct hydrogen peroxide into those cells
Significant SNP	Ch3: 21625003	AT3G58460		RHOMBOID-like protein 15 (RBL15)

Significant SNP	Ch3: 2231603	AT3G07050		GTP-binding family protein
Significant SNP	Ch3: 6968031	AT3G20000	translocase of the outer mitochondrial membrane 40 (*TOM40*)	Encodes a component of the TOM receptor complex responsible for the recognition and translocation of cytosolically synthesized mitochondrial preproteins. With TOM22, functions as the transit peptide receptor at the surface of the mitochondrial outer membrane and facilitates the movement of preproteins into the translocation pore.
Significant SNP	Ch3: 8014458	AT3G22640		PAP85
Significant SNP	Ch4: 5180340	AT4G08200		Similar to unknown protein [*Arabidopsis thaliana*] (TAIR:AT1G43722.1)
Significant SNP	Ch4: 6805259	AT4G11160		Translation initiation factor 2, small GTP-binding protein
Significant SNP	Ch4: 8654778	AT4G15180		SET domain protein 2 (SDG2)
Significant SNP	Ch4: 9350941	AT4G16600		Nucleotide-diphospho-sugar transferases superfamily protein
Significant SNP	Ch4:13265656	AT4G26190		Haloacid dehalogenase-like hydrolase (HAD) superfamily protein
Significant SNP	Ch4:13955847	AT4G28080		Tetratricopeptide repeat (TPR)-like superfamily protein
Significant SNP	Ch4:16420532	AT4G34320		Protein of unknown function (DUF677)
Significant SNP	Ch5: 22041081	AT5G54280	myosin 2 (*ATM2*)	Type VII myosin gene
Significant SNP	Ch5: 22677563	AT5G56000		HEAT SHOCK PROTEIN 81.4 (Hsp81.4)
Significant SNP	Ch5: 22842831	AT5G56390		F-box/RNI-like/FBD-like domains-containing protein
Significant SNP	Ch5: 23302987	AT5G57535		unknown protein
Significant SNP	Ch5: 414050	AT5G02100	Unfertilized embryo sac 18 (*UNE18*)	Encodes a protein that binds to beta-sitosterol and localizes to the ER. The WFDE motif in ORP3a appears to be important for a direct interaction with PVA12 [Plant VAMP-Associated protein 12]. Mutation of this motif causes ORP3a to relocalize to the Golgi and cytosol. The interaction between PVA12 and ORP3a does not appear to be sterol-dependent

Significant SNP	Ch5: 7493620	AT5G22560		Plant protein of unknown function (DUF247)
Significant SNP	Ch5: 7493623	AT5G23480		SWIB/MDM2 domain
Significant SNP	Ch5: 9154579	AT5G26190		Cysteine/Histidine-rich C1 domain family protein
in_LD_with	Ch1: 25500708	AT1G67990	*TSM1*	Encodes a tapetum-specific O-methyltransferase. In vitro enzyme assay indicated activity with caffeoyl-CoA, caffeoyl glucose, chlorogenic acid and polyamine conjugates. RNAi mutants had impaired silique development and seed setting.
in_LD_with	Ch1: 25500708	AT1G68010	hydroxypyruvate reductase (*HPR*)	Encodes hydroxypyruvate reductase.
in_LD_with	Ch1: 25500708	AT1G67980	caffeoyl-CoA 3-O-methyltransferase (*CCOAMT*)	Encodes S-adenosyl-L-methionine: transcaffeoyl Coenzyme A 3-O-methyltransferase.
in_LD_with	Ch1: 25500708	AT1G67960		CONTAINS InterPro DOMAIN/s: Membrane protein,Tapt1/CMV receptor (InterPro:IPR008010)
in_LD_with	Ch1: 25500708	AT1G68000	phosphatidylinositol synthase 1 (*PIS1*)	phosphatidylinositol synthase 1
in_LD_with	Ch1: 25500708	AT1G68020	*ATTPS6*	Encodes an enzyme putatively involved in trehalose biosynthesis. The protein has a trehalose synthase (TPS)-like domain and a trehalose phosphatase (TPP)-like domain. It can complement a yeast mutant lacking both of these activities suggesting that this is a bifunctional enzyme.
in_LD_with	Ch1: 25500708	AT1G67970	heat shock transcription factor A8 (*HSFA8*)	member of Heat Stress Transcription Factor (Hsf) family
in_lD_with	Ch1: 29518622	AT1G78440	*Arabidopsis thaliana* gibberellin 2-oxidase 1 (*ATGA2OX1*)	Encodes a gibberellin 2-oxidase that acts on C19 gibberellins.
in_lD_with	Ch1: 29518622	AT1G78430		ROP interactive partner 2 (RIP2)

in_lD_with	Ch1: 29518622	AT1G78450		SOUL heme-binding family protein
in_lD_with	Ch1: 29518622	AT1G78470		BEST Arabidopsis thaliana protein match is: F-box family protein (TAIR:AT1G67390.1)
in_LD_with	Ch1: 7207918	AT1G20740		Protein of unknown function (DUF833)
in_LD_with	Ch1: 7207918	AT1G20760		Calcium-binding EF hand family protein
in_LD_with	Ch1: 7207918	AT1G20780	senescence-associated E3 ubiquitin ligase 1 (*SAUL1*)	Encodes a protein containing a U-box and an ARM domain.
in_LD_with	Ch1: 7207918	AT1G20790		F-box family protein
in_LD_with	Ch1: 7207918	AT1G20770		Unknown protein
in_LD_with	Ch2: 11659416	AT2G27250	*AtCLV3*	One of the three CLAVATA genes controlling the size of the shoot apical meristem (SAM) in *Arabidopsis*. Belongs to a large gene family called CLE for CLAVATA3/ESR-related. Encodes a stem cell-specific protein CLV3 presumed to be a precursor of a secreted peptide hormone. The deduced ORF encodes a 96-amino acid protein with an 18-amino acid N-terminal signal peptide. The functional form of CLV3 (MCLV3) was first reported to be a posttranscriptionally modified 12-amino acid peptide, in which two of the three prolines were modified to hydroxyproline
in_LD_with	Ch3: 19804402	AT3G53400		BEST Arabidopsis thaliana protein match is: conserved peptide upstream open reading frame 47 (TAIR:AT5G03190.1)
in_LD_with	Ch3: 21625003	AT3G53410		RING/U-box superfamily protein
in_LD_with	Ch3: 21625003	AT3G58490		Phosphatidic acid phosphatase (PAP2) family protein
in_LD_with	Ch3: 21625003	AT3G58450		Adenine nucleotide alpha hydrolases-like superfamily protein
in_LD_with	Ch3: 21625003	AT3G58510		DEA(D/H)-box RNA helicase family protein
in_LD_with	Ch3: 21625003	AT3G58440		TRAF-like superfamily protein

in_LD_with	Ch3: 21625003	AT3G58520		Ubiquitin carboxyl-terminal hydrolase family protein
in_LD_with	Ch3: 21625003	AT3G58480		Calmodulin-binding family protein
in_LD_with	Ch3: 21625003	AT3G58470		Nucleic acid binding
in_LD_with	Ch3: 21625003	AT3G58500		Encodes one of the isoforms of the catalytic subunit of protein phosphatase 2A: AT1G59830/PP2A-1, AT1G10430/PP2A-2, At2g42500/PP2A-3, At3g58500/PP2A-4 [Plant Molecular Biology (1993) 21:475-485 and (1994) 26:523-528
in_LD_with	Ch3: 6968031	AT3G20010		SNF2 domain-containing protein / helicase domain-containing protein / zinc finger protein-related
in_LD_with	Ch3: 6968031	AT3G19990		Unknown protein
in_LD_with	Ch3: 6968031	AT3G19980		Encodes catalytic subunit of serine/threonine protein phosphatase 2A. It can associate with phytochromes A and B in vitro. Mutant plants display an accelerated flowering phenotype.
in_LD_with	Ch3: 8014458	AT3G22670		Pentatricopeptide repeat (PPR) superfamily protein
in_LD_with	Ch3: 8014458	AT3G22680	RNA-directed DNA methylation 1 (RDM1)	Encodes RNA-DIRECTED DNA METHYLATION 1 (RDM1), forming a complex with DMS3 (AT3G49250) and DRD1 (AT2G16390). This complex is termed DDR. The DDR complex is required for polymerase V transcripts and RNA-directed DNA methylation.
in_LD_with	Ch3: 8014458	AT3G22650		CEGENDUO (CEG)
in_LD_with	Ch3: 8014458	AT3G22690		Involved in: photosystem II assembly, regulation of chlorophyll biosynthetic process, photosystem I assembly, thylakoid membrane organization, RNA modification
in_LD_with	Ch3: 8014458	AT3G22700		F-box and associated interaction domains-containing protein
in_LD_with	Ch3: 8014458	AT3G22710		F-box family protein

in_LD_with	Ch3: 8014458	AT3G22720		F-box and associated interaction domains-containing protein
in_LD_with	Ch3: 8014458	AT3G22730		F-box and associated interaction domains-containing protein
in_LD_with	Ch3: 8014458	AT3G22740	homocysteine S-methyltransferase 3 (*HMT3*)	Homocysteine S-methyltransferase (HMT3)
in_LD_with	Ch3: 8014458	AT3G22750		Protein kinase superfamily protein
in_LD_with	Ch3: 8014458	AT3G22760	*SOL1*	CXC domain containing TSO1-like protein 1. The gene is expressed in stamens, pollen mother cells, and immature ovules.
in_LD_with	Ch4: 5180340	AT4G08190		P-loop containing nucleoside triphosphate hydrolases superfamily protein
in_LD_with	Ch4: 5180340	AT4G08180		OSBP(oxysterol binding protein)-related protein 1C (ORP1C)
in_LD_with	Ch4: 5180340	AT4G08230		Glycine-rich protein
in_LD_with	Ch4: 5180340	AT4G08210		Pentatricopeptide repeat (PPR-like) superfamily protein
in_LD_with	Ch4: 5180340	AT4G08220		Mutator-like transposase family, has a $5.3*10^{-67}$ P-value blast match to Q9SUF8 /145-308 Pfam PF03108 MuDR family transposase (MuDr-element domain)
in_LD_with	Ch4: 6805259	AT4G11140	cytokinin response factor 1 (*CRF1*)	Encodes a member of the ERF (ethylene response factor) subfamily B-5 of the ERF/AP2 transcription factor family. The protein contains one AP2 domain. There are seven members in this subfamily. Also named as CRF1 (cytokinin response factor 1).
in_LD_with	Ch4: 6805259	AT4G11150	vacuolar ATP synthase subunit E1 (*TUF*)	Encodes a vacuolar H^+-ATPase subunit E isoform 1 which is required for Golgi organization and vacuole function in embryogenesis.
in_LD_with	Ch4: 6805259	AT4G11170	*RMG1*	Disease resistance protein (TIR-NB-LRR class) family
in_LD_with	Ch4: 8654778	AT4G15210	*Arabidopsis thaliana* BETA-AMYLASE (*ATBETA-*	Cytosolic beta-amylase expressed in rosette leaves and inducible by sugar. RAM1 mutants have reduced beta

			AMY)	amylase in leaves and stems.
in_LD_with	Ch4:13265656	AT4G26180		Mitochondrial substrate carrier family protein
in_LD_with	Ch4:13265656	AT4G26150	cytokinin-responsive gata factor 1 (*CGA1*)	Encodes a member of the GATA factor family of zinc finger transcription factors.
in_LD_with	Ch4:13265656	AT4G26170		Molecular_function unknown
in_LD_with	Ch4:13265656	AT4G26220		S-adenosyl-L-methionine-dependent methyltransferases superfamily protein
in_LD_with	Ch4:13265656	AT4G26140	beta-galactosidase 12 (BGAL12)	Putative beta-galactosidase
in_LD_with	Ch4:13265656	AT4G26160	atypical CYS HIS rich thioredoxin 1 (*ACHT1*)	Encodes a member of the thioredoxin family protein. Located in the chloroplast. Shows high activity towards the chloroplast 2-Cys peroxiredoxin A, and poor activity towards the chloroplast NADP-malate dehydrogenase
in_LD_with	Ch4:13265656	AT4G26210		Mitochondrial ATP synthase subunit G protein
in_LD_with	Ch4:13955847	AT4G26200	1-amino-cyclopropane-1-carboxylate synthase 7 (*ACS7*)	Member of a family of proteins in *Arabidopsis* that encode 1-Amino-cyclopropane-1-carboxylate synthase, an enzyme involved in ethylene biosynthesis. Not expressed in response to IAA
in_LD_with	Ch4:13955847	AT4G28100		Unknown protein
in_LD_with	Ch4:13955847	AT4G28060		Cytochrome c oxidase, subunit Vib family protein
in_LD_with	Ch4:13955847	AT4G28070		AFG1-like ATPase family protein
in_LD_with	Ch4:13955847	AT4G28090		SKU5 similar 10 (sks10)
in_LD_with	Ch4:13955847	AT4G28085		Unknown protein
in_LD_with	Ch4:13955847	AT4G28088		Low temperature and salt responsive protein family
in_LD_with	Ch4:13955847	AT4G34310		alpha/beta-Hydrolases superfamily protein

in_LD_with	Ch5: 22041081	AT5G54250	cyclic nucleotide-gated cation channel 4 (*CNGC4*)	Member of Cyclic nucleotide gated channel family, downstream component of the signaling pathways leading to HR resistance. Mutant plants exhibit gene-for-gene disease resistance against avirulent *Pseudomonas syringae* despite the near-complete absence of the hypersensitive response (HR). Salicylic acid accumulation in dnd2 mutants is completely PAD4-independent.
in_LD_with	Ch5: 22041081	AT5G54260	Meiotic recombination 11 (*MRE11*)	DNA repair and meiotic recombination protein, component of MRE11 complex with RAD50 and NBS1
in_LD_with	Ch5: 22041081	AT5G54270	light-harvesting chlorophyll B-binding protein 3 (*LHCB3*)	Lhcb3 protein is a component of the main light harvesting chlorophyll a/b-protein complex of Photosystem II (LHC II).
in_LD_with	Ch5: 22041081	AT5G54240		Protein of unknown function (DUF1223)
in_LD_with	Ch5: 22677563	AT5G55990	calcineurin B-like protein 2 (*CBL2*)	Encodes a member of the *Arabidopsis* CBL (Calcineurin B-like Calcium Sensor) protein family
in_LD_with	Ch5: 22677563	AT5G55980		Serine-rich protein-related
in_LD_with	Ch5: 22677563	AT5G55970		RING/U-box superfamily protein
in_LD_with	Ch5: 22842831	AT5G56380		F-box/RNI-like/FBD-like domains-containing protein
in_LD_with	Ch5: 22842831	AT5G56370		F-box/RNI-like/FBD-like domains-containing protein
in_LD_with	Ch5: 22842831	AT5G56368		Encodes a defensin-like (DEFL) family protein.
in_LD_with	Ch5: 23302987	AT5G57520	zinc finger protein 2 (*ZFP2*)	Encodes a zinc finger protein containing only a single zinc finger.
in_LD_with	Ch5: 23302987	AT5G57560	Touch 4 (*TCH4*)	Encodes a cell wall-modifying enzyme, rapidly upregulated in response to environmental stimuli

in_LD_with	Ch5: 23302987	AT5G57490	voltage dependent anion channel 4 (*VDAC4*)	Encodes a voltage-dependent anion channel (VDAC: AT3G01280/VDAC1, AT5G67500/VDAC2, AT5G15090/VDAC3, AT5G57490/VDAC4, AT5G15090/VDAC5). VDACs are reported to be porin-type, beta-barrel diffusion pores. They are prominently localized in the outer mitochondrial membrane and are involved in metabolite exchange between the organelle and the cytosol.
in_LD_with	Ch5: 23302987	AT5G57565		Protein kinase superfamily protein
in_LD_with	Ch5: 23302987	AT5G57540		Encodes a xyloglucan endotransglucosylase/hydrolase with only only the endotransglucosylase (XET
in_LD_with	Ch5: 23302987	AT5G57550	xyloglucan endotransglucosylase/hydrolase 25 (*XTH25*)	Xyloglucan endotransglycosylase-related protein (XTR3)
in_LD_with	Ch5: 23302987	AT5G57500		Galactosyltransferase family protein
in_LD_with	Ch5: 23302987	AT5G57530		Xyloglucan endotransglucosylase/hydrolase 12 (XTH12)
in_LD_with	Ch5: 23302987	AT5G57510		Unknown protein
in_LD_with	Ch5: 23302987	AT5G57570		GCK domain-containing protein
in_LD_with	Ch5: 23302987	AT5G57590	biotin auxotroph 1 (*BIO1*)	Mutant complemented by E coli Bio A gene encoding 7,8-diaminopelargonic acid aminotransferase.
in_LD_with	Ch5: 23302987	AT5G57580		Calmodulin-binding protein
in_LD_with	Ch5: 414050	AT5G02110		CYCLIN D7
in_LD_with	Ch5: 7493620	AT5G22550		Plant protein of unknown function (DUF247)
in_LD_with	Ch5: 7493620	AT5G22570	*WRKY38*	member of WRKY Transcription Factor
in_LD_with	Ch5: 7493620	AT5G22545		Unknown protein
in_LD_with	Ch5: 7493620	AT5G22555		Unknown protein
in_LD_with	Ch5: 7493623	AT5G23510		Unknown protein
in_LD_with	Ch5: 7493623	AT5G23490		Unknown protein

Table S4 Genes in linkage with SNPs with –$\log_{10}$ (*P*) score above 4 (20 kb half-window size) in the contrast-specific GWA mapping of parasitic plants and aphids on the one hand vs fungus, caterpillar, thrips and drought on the other hand

Marker	Gene in LD	Gene name	Gene description	Responsiveness	Reference
chr1.19711816	*AT1G52900*	-	Toll-Interleukin-Resistance (TIR) domain family protein, signal transduction, defense response	Pseudomonas	(Cartieaux *et al.*, 2008)
chr1.24785939	*AT1G66410*	*CAM4*	Calmodulin 4, calcium-binding EF-hand site, calcium-mediated signalling	unknown	(Zhao *et al.*, 2013)
chr3.672138	*AT3G02940*	*MYB107*	Transcription factor, responsive to salicylic acid	SA	(Stracke *et al.*, 2001; Yanhui *et al.*, 2006)
chr3.7945317	*AT3G22400*	*LOX5*	Oxidoreductase activity (9-LOX pathway), facilitates *M. persicae* aphid feeding	aphids	(Nalam *et al.*, 2012a; Nalam *et al.*, 2012b)
chr3.23145919	*AT3G62610*	*MYB11*	Transcription factor, involved in production of flavonol glycosides	unknown	(Stracke *et al.*, 2007)
chr4.9390514	*AT4G16730*, *AT4G16740*,	*TPS02*, *TPS03*	Terpene synthases, (*E,E*)-alpha-farnesene synthase	Salt, resp. heat, high light	(Huang *et al.*, 2010; Rasmussen *et al.*, 2013)
	AT4G16690	*MES16*	Methyl jasmonate esterase	unknown	(Christ *et al.*, 2012)
chr5.22829754	*AT5G56360*	*PSL4*	Calmodulin binding protein, involved in MAMP-triggered defense to bacteria	MAMP	(Lu *et al.*, 2009)

Table S5 Candidate genes in linkage with SNPs with –$\log_{10}$(P) score above 4 (20 kb half-window size) that have common effects on plant response to parasitic plants and aphids on the one hand vs fungus, caterpillar, thrips and drought on the other hand

Marker	Gene	Gene name	Description	Responsiveness	References
chr2.15762021	*AT2G37570*	*SLT1*	Encodes a protein that can complement the salt-sensitive phenotype of a calcineurin (CaN)-deficient yeast mutant.	Unknown	(Matsumoto *et al.*, 2001)
in_LD_with_chr2.15762021	*AT2G37630*	*MYB91*	Encodes a MYB-domain protein involved in specification of the leaf proximodistal axis. Also functions as a regulator of the plant immune response.	Necrotrophic fungi, auxin	(Nurmberg *et al.*, 2007)
in_LD_with_chr3.22345759	*AT3G60490*	-	Encodes a member of the DREB subfamily A-4 of ERF/AP2 transcription factor family. Pathogenesis-related.	Pathogens	(Mitchell *et al.*, 2015)
chr4.9598560	*AT4G17070*	-	Encodes a peptidyl-prolyl cis-trans isomerase. Involved in response to oxidative stress.	Oxidative stress, salinity, osmotic stress	(Luhua *et al.*, 2008)

Table S6 Candidate genes in linkage with SNPs with $-\log_{10}(P)$ score above 4 (20 kb half-window size) that have common effects on biotic and abiotic stress responses

Marker	Gene	Gene name	Description	Responsiveness	References
in_LD_with_chr4.5651749	*AT4G08870*	*ARGAH2*	Encodes one of the two arginases in the genome. Gene expression is enhanced by methyl jasmonate treatment. It is involved in the defence response to *B. cinerea*.	JA, bio- and necrotrophic pathogens, salt, high light	(Jubault *et al.*, 2008; Gravot *et al.*, 2012; Rasmussen *et al.*, 2013)
chr4. 8057710	*AT4G13940*	*AtSAHH1*	Encodes an S-adenosyl-L-homocysteine hydrolase required for DNA methylation-dependent gene silencing.	Heat stress	(Min *et al.*, 2014)
chr2.856085	*AT2G02950*	*PKS1*	Encodes a basic soluble protein which can independently bind to either PHYA or PHYB, regardless of whether the phytochromes are in the Pr or Pfr state. PKS1 can be phosphorylated by oat phyA *in vitro* in a light-regulated manner. It is postulated to be a negative regulator of phyB signalling.	Light	(Fankhauser *et al.*, 1999; Molas and Kiss, 2008)

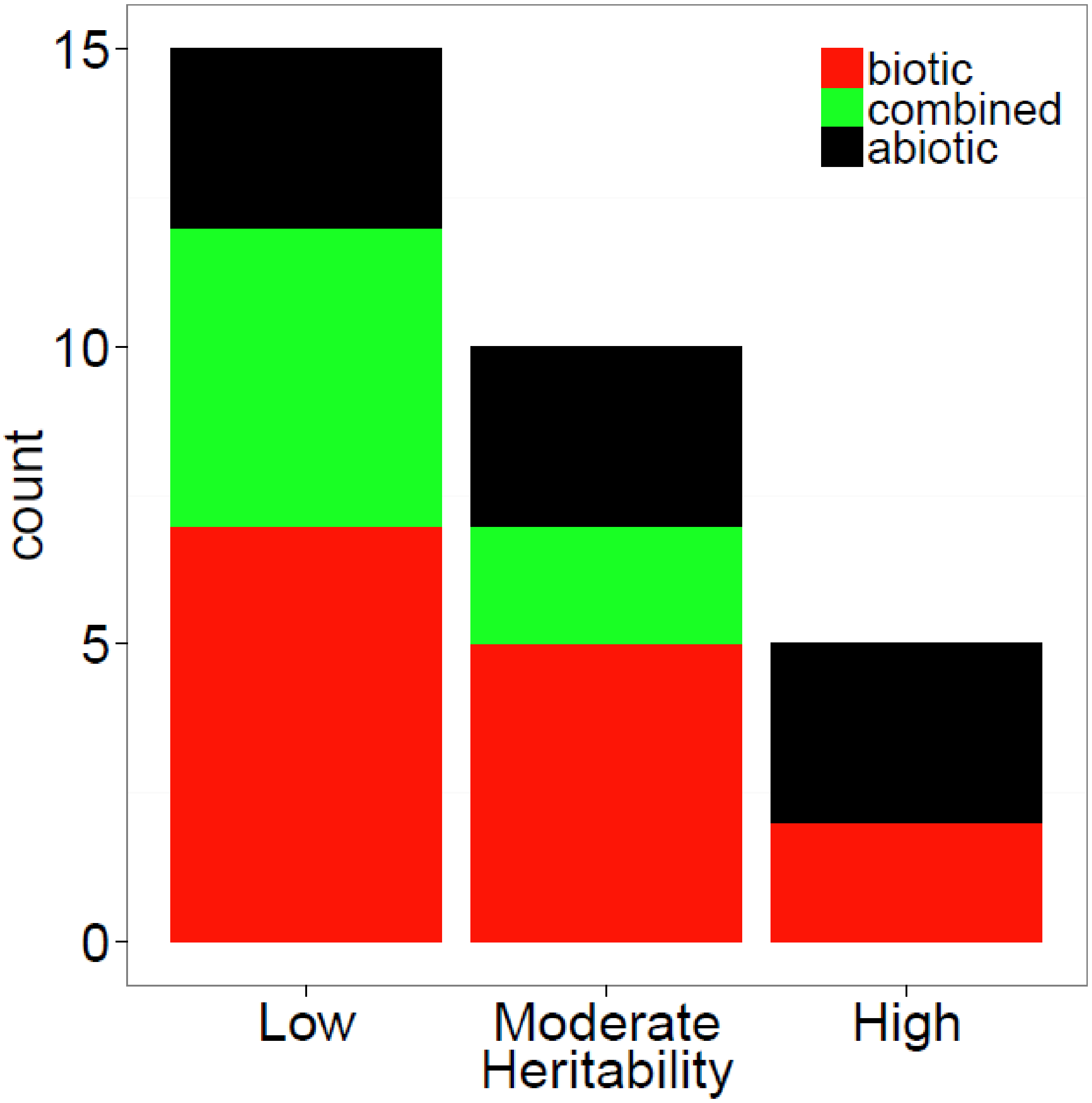

Fig. S1 Narrow sense heritability for *Arabidopsis thaliana* resistance to abiotic and biotic stresses. Narrow sense heritability values were estimated using the 'heritability' R package. Traits were classified in three biological categories: resistance to abiotic, biotic and double stresses. These biological categories were grouped based on their heritability in low (h^2<0.2), moderate (0.2<h^2<0.5) and high (h^2>0.5) heritability classes.

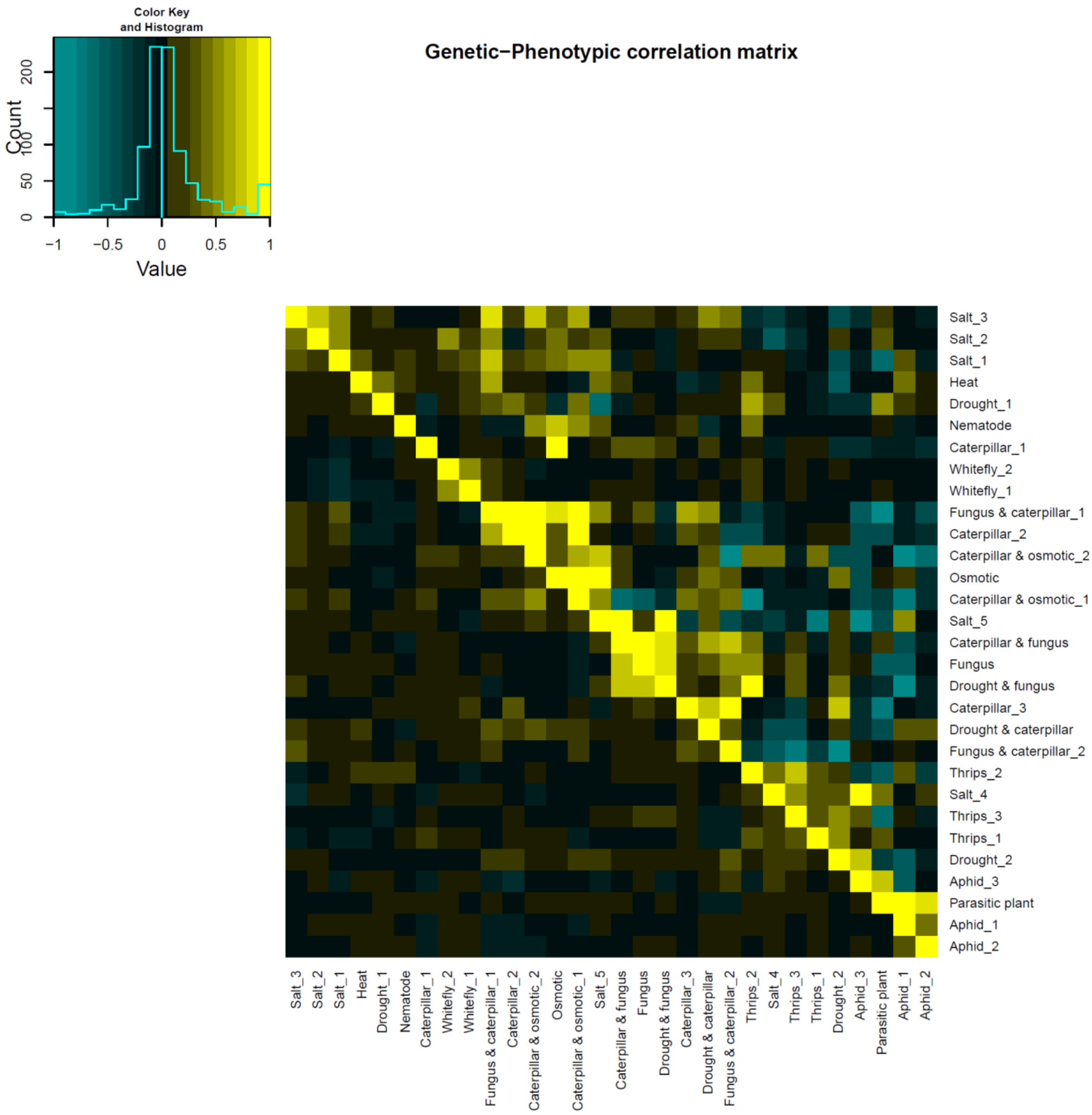

Fig. S2 Genetic and phenotypic correlation matrix. Heatmap displaying phenotypic correlations below the diagonal and genetic correlations above the diagonal. The genetic correlations shown above the diagonal are the same ones as those used for the construction of Fig. 1 in the main text. Phenotypic correlations were calcluated using Spearman's correlation coefficient *rho*, whereas the genome-wide genetic correlations were estimated bivariately and with correction for population structure (on full kinship matrix). For Whitefly_1 and Whitefly_2 the maximum likelihood estimates were not available so genetic correlations were estimated using G-BLUP. Traits were clustered according to Ward's minimum variance method for the genetic correlation coefficient values.

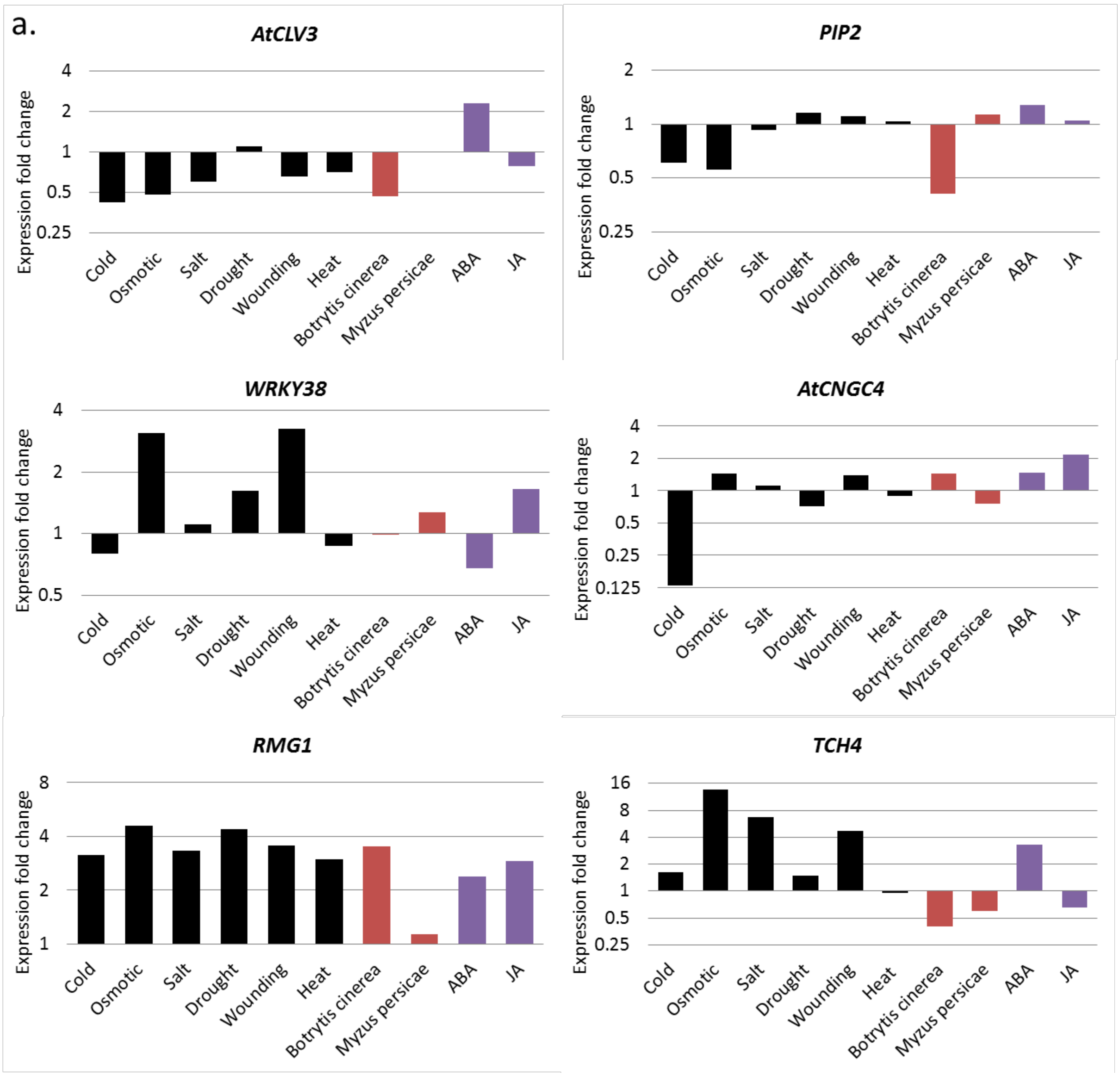
a.
AtCLV3
Expression fold change
4
2
1
0.5
0.25
Cold
Osmotic
Salt
Drought
Wounding
Heat
Botrytis cinerea
Myzus persicae
ABA
JA
PIP2
Expression fold change
2
1
0.5
0.25
Cold
Osmotic
Salt
Drought
Wounding
Heat
Botrytis cinerea
Myzus persicae
ABA
JA
WRKY38
Expression fold change
4
2
1
0.5
Cold
Osmotic
Salt
Drought
Wounding
Heat
Botrytis cinerea
Myzus persicae
ABA
JA
AtCNGC4
Expression fold change
4
2
1
0.5
0.25
0.125
Cold
Osmotic
Salt
Drought
Wounding
Heat
Botrytis cinerea
Myzus persicae
ABA
JA
RMG1
Expression fold change
8
4
2
1
Cold
Osmotic
Salt
Drought
Wounding
Heat
Botrytis cinerea
Myzus persicae
ABA
JA
TCH4
Expression fold change
16
8
4
2
1
0.5
0.25
Cold
Osmotic
Salt
Drought
Wounding
Heat
Botrytis cinerea
Myzus persicae
ABA
JA

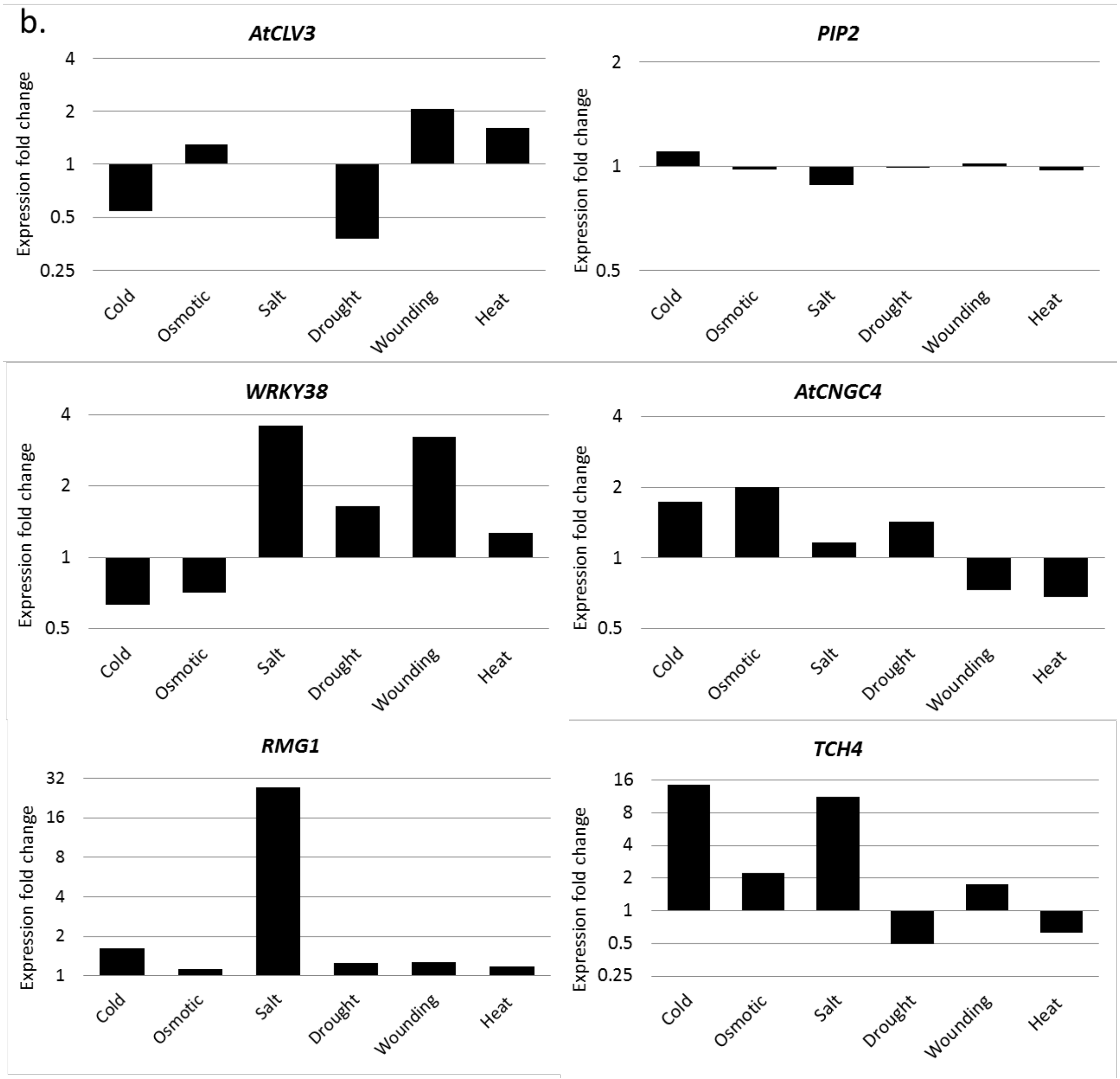

Fig. S3 Expression data of six candidate genes (resulting from MTMM, see Table 2a) in plants exposed to biotic or abiotic stress factors, relative to control conditions. (a) Shoot tissues and (b) root tissues. Black bars represent abiotic stresses, red bars represent biotic stresses and purple bars represent phytohormonal treatments. Expression data from Arabidopsis eFP browser (http://bbc.botany.utoronto.ca).

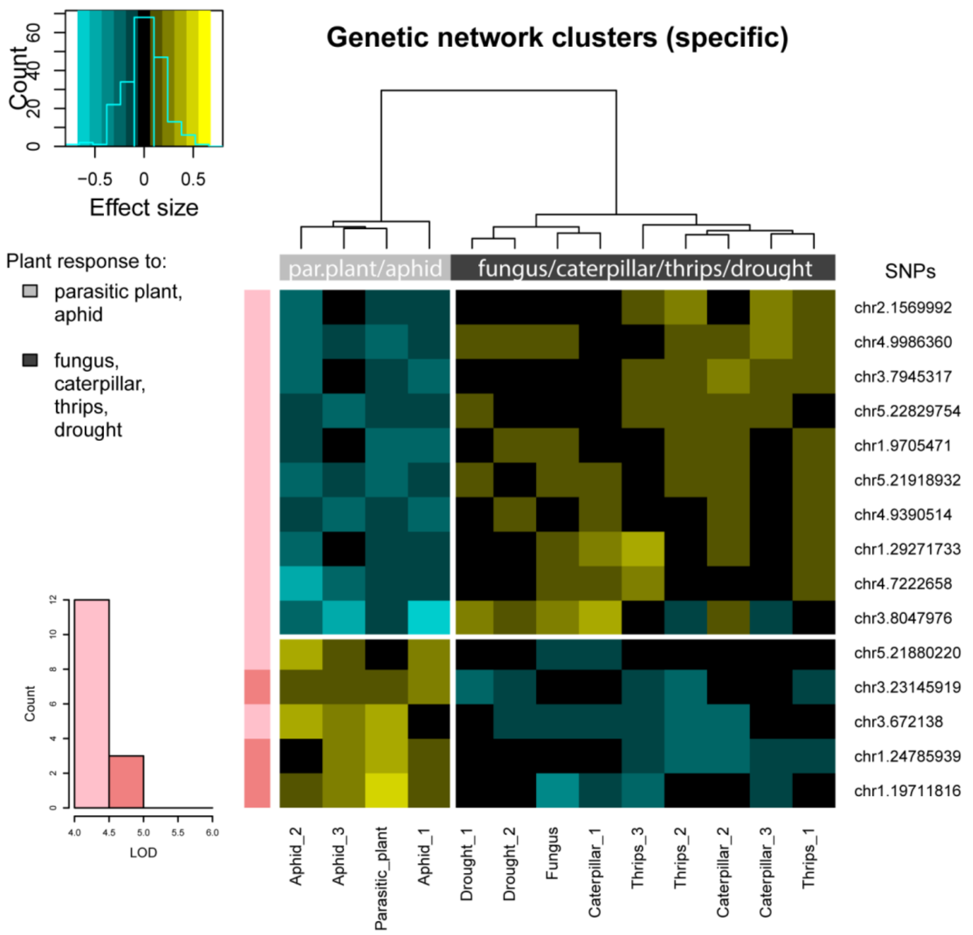

Fig. S4 Genetic associations specific for plant responses to the main clusters of the genetic correlation network (see Fig. 1): parasitic plant and aphid vs fungus, caterpillar, thrips and drought. Genetic associations were estimated with a contrast-specific analysis using MTMM. Significant SNPs ($P \leq 10^{-4}$) for the contrast are clustered according to trait-specific effects estimated from the full MTMM. If there was another SNP in LD that had a higher effect size, this SNP was used as representative for the LD block. Negative effect sizes (blue) were cases where the rare allele was associated with a detrimental effect on the plants, positive effect sizes (yellow) were cases where the rare allele was associated with increased resistance to the stress. The rare alleles of the top 10 SNPs are associated with enhanced resistance to **fungus, caterpillar, thrips and drought** stresses and reduced resistance to stresses inflicted by **parasitic plants and aphids**; the bottom 5 SNPs show the inverse. Stresses are clustered according to effect size, using Ward's minimum variance method. If SNPs were located within a 20 kb half-window of each other, only the SNP with the highest absolute cumulative effect size was included. The key shows the frequency distribution of SNPs across effect sizes.

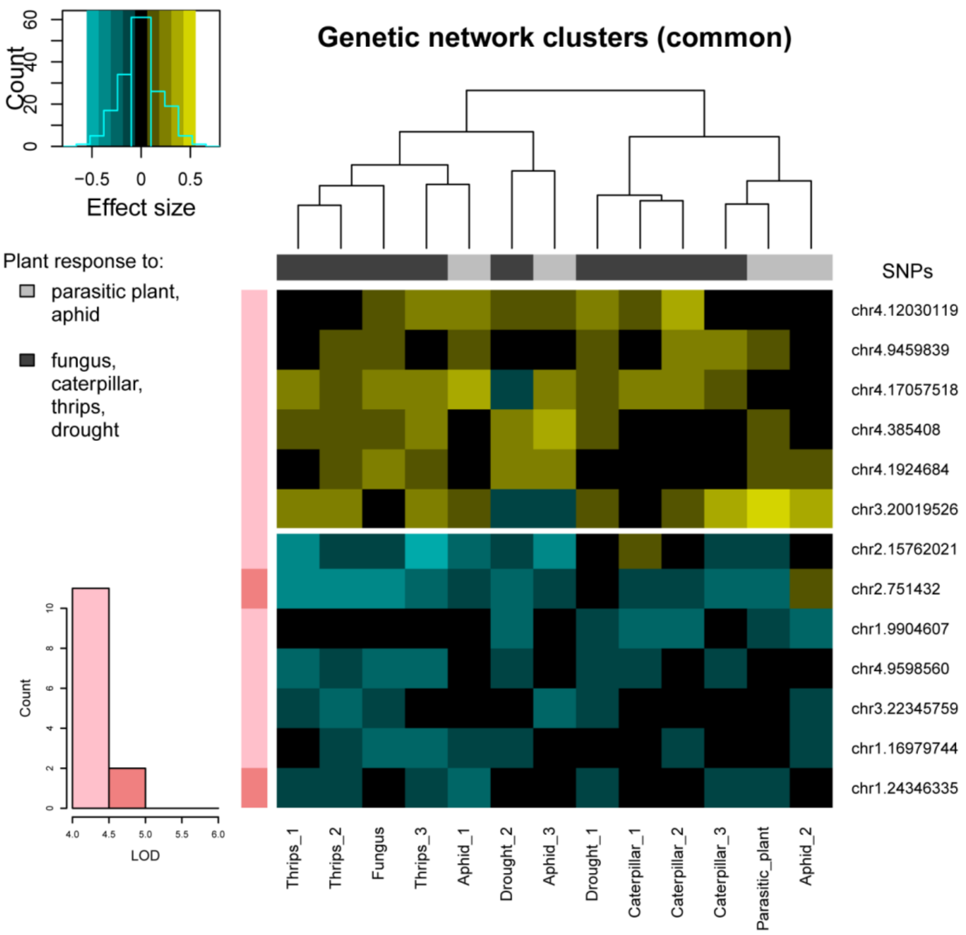

Fig. S5 Genetic associations common for plant response to the main clusters of the genetic correlation network: parasitic plant and aphid on the one hand vs fungus, caterpillar, thrips and drought on the other hand. Genetic associations were estimated with a contrast analysis using MTMM. Significant SNPs ($P \leq 10^{-4}$) for the common response are clustered according to trait-specific effects estimated from the full MTMM. If there was another SNP in LD that had a higher effect size, this SNP was used as representative for the LD block. Negative effect sizes (blue) were cases where the rare allele was associated with a detrimental effect on the plants, positive effect sizes (yellow) were cases where the rare allele was associated with increased resistance to the stress. The rare alleles of the top 6 SNPs are associated with enhanced resistance to abiotic stresses and reduced resistance to biotic stresses; the bottom 7 SNPs show the inverse. Stresses are clustered according to SNP effect size, using Ward's minimum variance method. If SNPs were located within a 20 kb half-window of each other, only the SNP with the highest absolute cumulative effect size was included. The key shows the frequency distribution of SNPs across effect sizes.

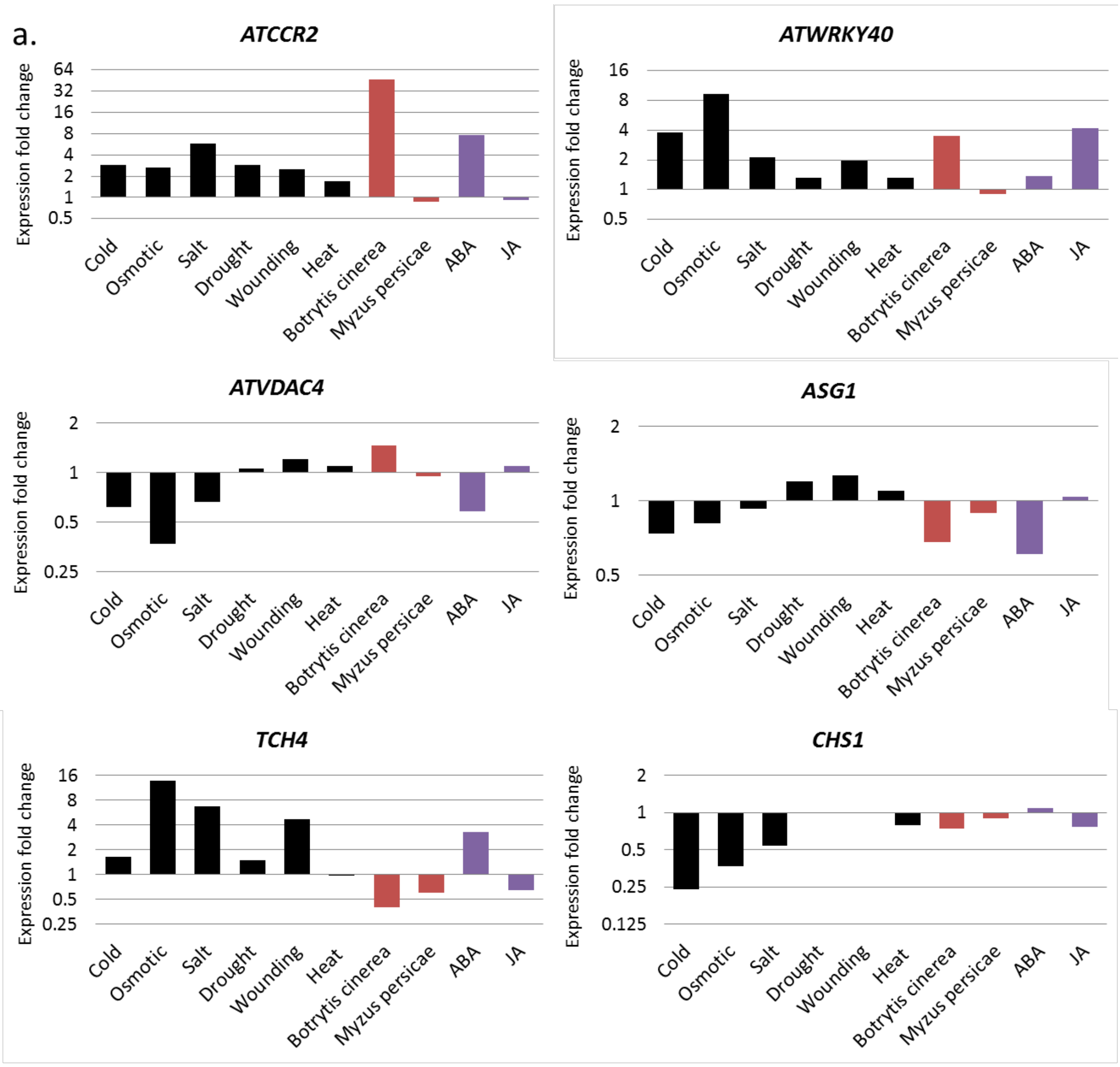
a.
ATCCR2
Expression fold change
64
32
16
8
4
2
1
0.5
Cold
Osmotic
Salt
Drought
Wounding
Heat
Botrytis cinerea
Myzus persicae
ABA
JA
ATWRKY40
Expression fold change
16
8
4
2
1
0.5
ATVDAC4
Expression fold change
2
1
0.5
0.25
ASG1
Expression fold change
2
1
0.5
TCH4
Expression fold change
16
8
4
2
1
0.5
0.25
CHS1
Expression fold change
2
1
0.5
0.25
0.125

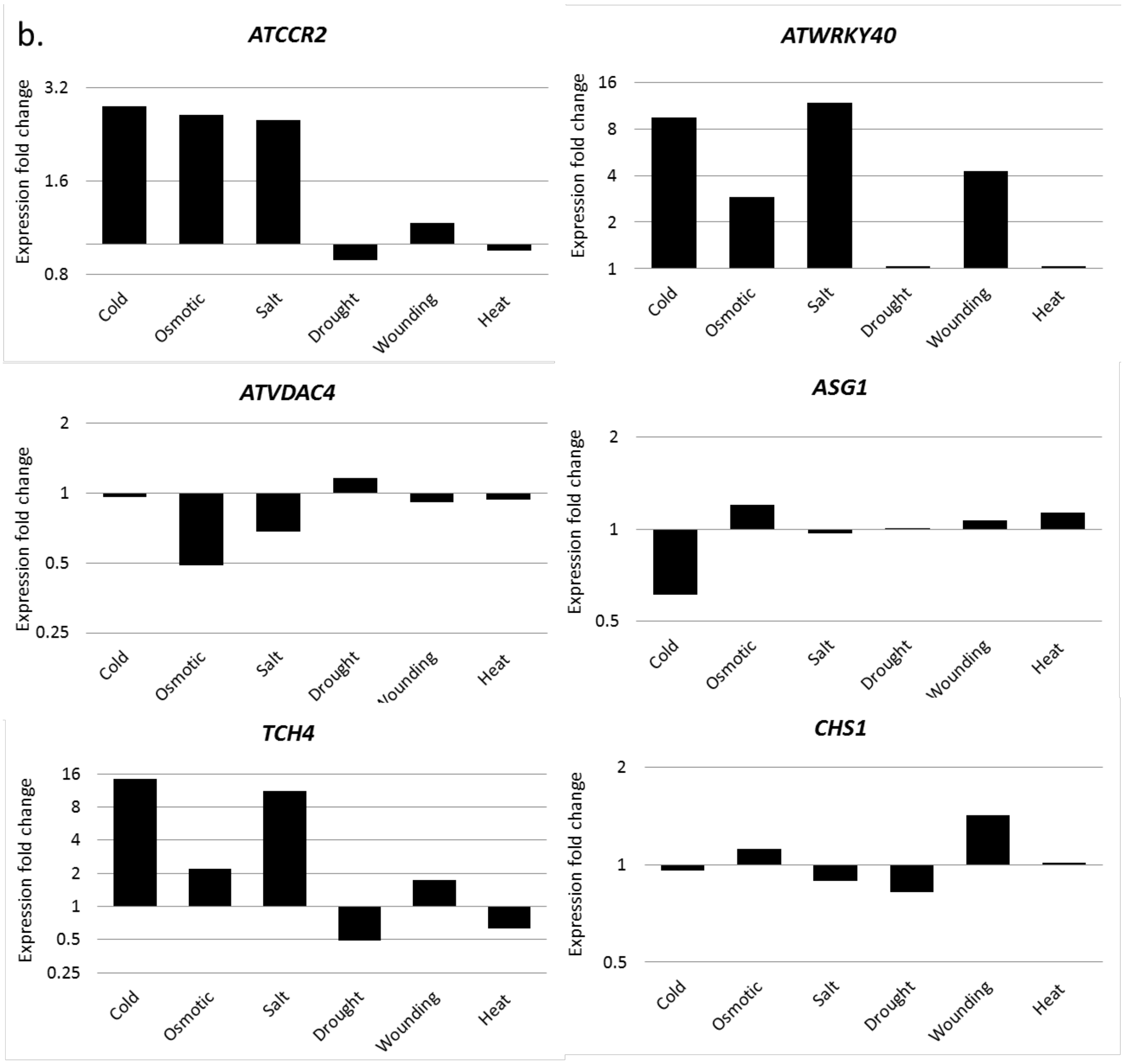

Fig. S6 Expression data of six candidate genes (resulting from MTMM analysis, see Table 2b) in plants exposed to biotic or abiotic stress factors, relative to control conditions. (a) Shoot tissues and (b) root tissues. Black bars represent abiotic stresses, red bars represent biotic stresses and purple bars represent phytohormonal treatments. Expression data from Arabidopsis eFP browser (http://bbc.botany.utoronto.ca).

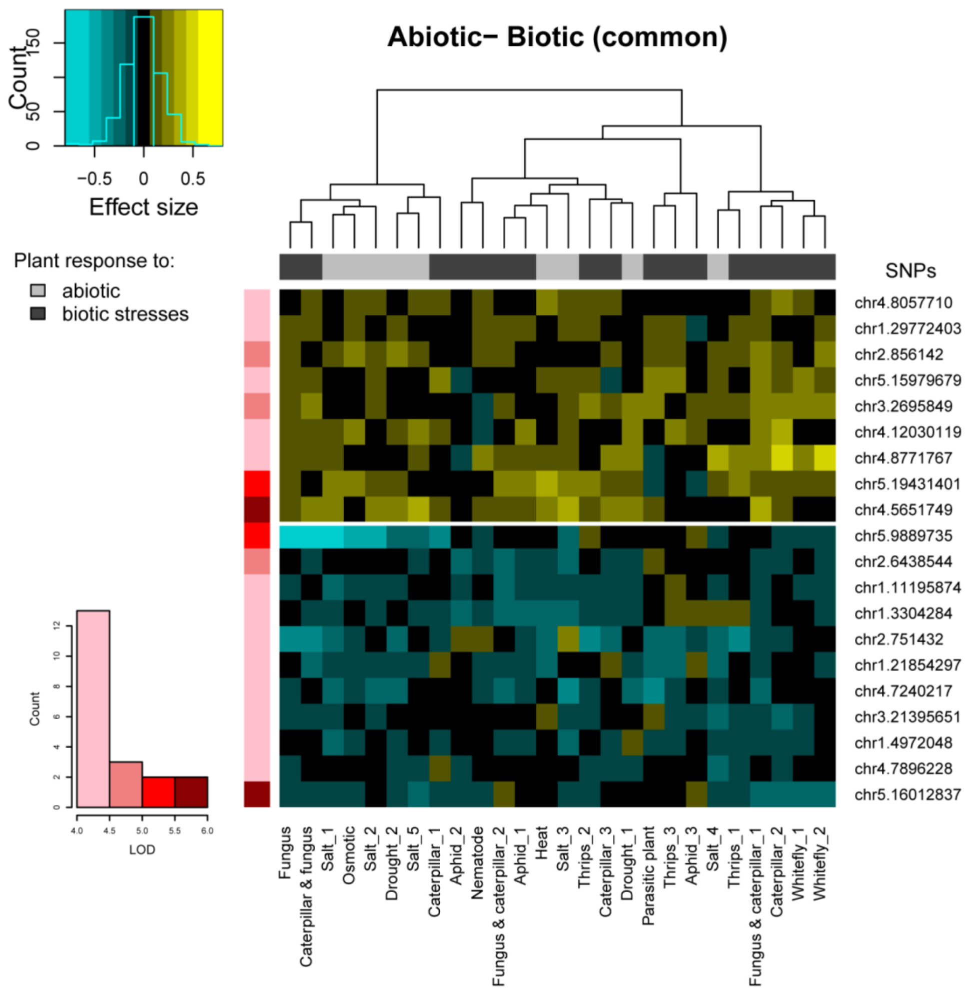

Fig. S7 Genetic associations common for plant responses to abiotic and biotic stresses. Genetic associations were estimated with a contrast analysis using MTMM. Significant SNPs ($P \leq 10^{-4}$) for the common response are clustered according to trait-specific effects estimated from the full MTMM. If there was another SNP in LD that had a higher effect size, this SNP was used as representative for the LD block. Negative effect sizes (blue) were cases where the rare allele was associated with a detrimental effect on the plants, positive effect sizes (yellow) were cases where the rare allele was associated with increased resistance to the stress. The rare alleles of the top 9 SNPs are associated with enhanced resistance to abiotic and biotic stresses; the bottom 11 SNPs are associated with reduced resistance to abiotic and biotic stresses. Stresses are clustered according to SNP effect size, using Ward's minimum variance method. If SNPs were located within a 20 kb half-window of each other, only the SNP with the highest absolute cumulative effect size was included. The key shows the frequency distribution of SNPs across effect sizes.

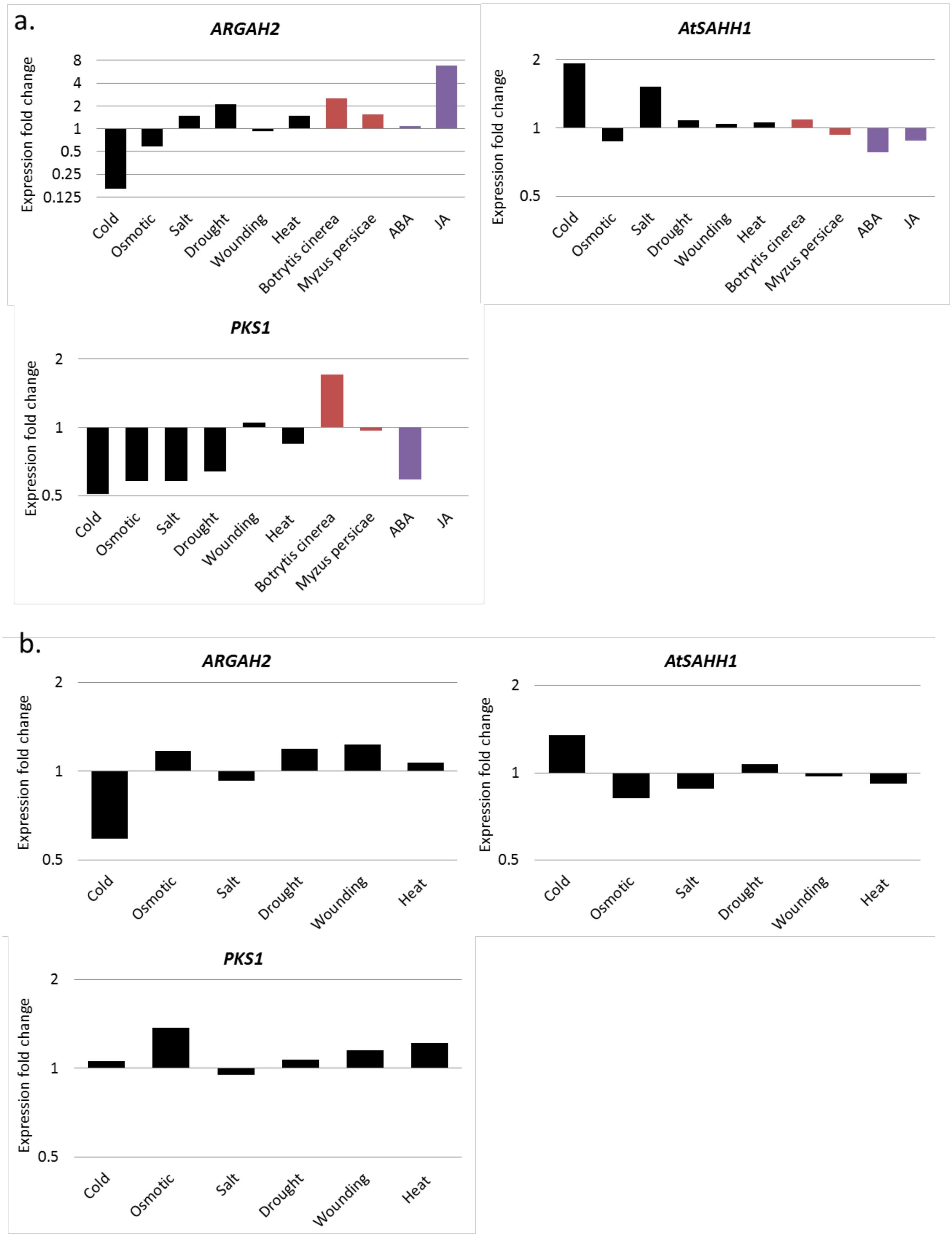

Fig. S8 Expression data of three candidate genes (resulting from MTMM, see Table S6) in plants exposed to biotic or abiotic stress factors, relative to control conditions. (a) Shoot tissues and (b) root tissues. Black bars represent abiotic stresses, red bars represent biotic stresses and purple bars represent phytohormonal treatments. Expression data from Arabidopsis eFP browser (http://bbc.botany.utoronto.ca).

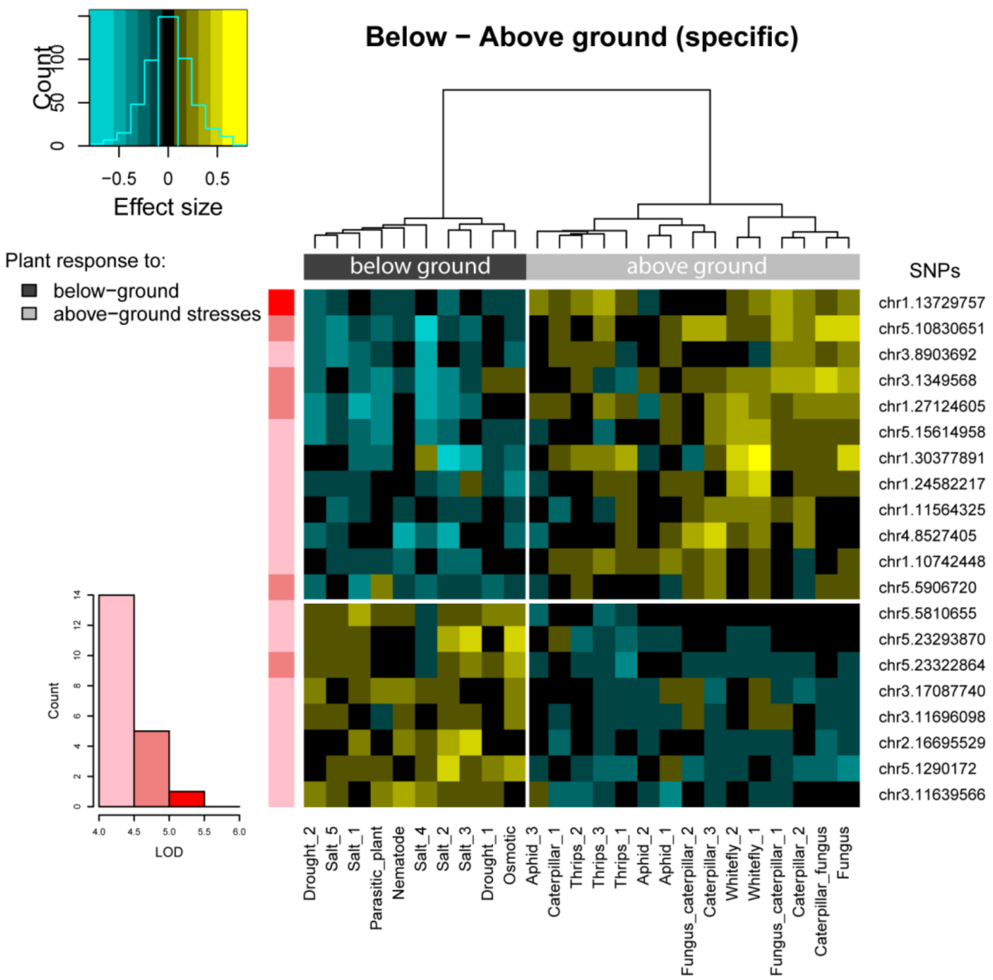

Fig. S9 Genetic associations specific for plant responses to either below- or aboveground stress. Genetic associations were estimated with a contrast analysis using MTMM. Significant SNPs ($P \leq 10^{-4}$) for the belowground-aboveground contrast are clustered according to trait-specific effects estimated from the full MTMM. If there was another SNP in LD that had a higher effect size, this SNP was used as representative for the LD block. Negative effect sizes (blue) were cases where the rare allele was associated with a detrimental effect on the plants, positive effect sizes (yellow) were cases where the rare allele was associated with increased resistance to the stress. The rare alleles of the top 12 SNPs are associated with enhanced resistance to aboveground stresses and reduced resistance to belowground stresses; the bottom 8 SNPs show the inverse. Stresses are clustered according to SNP effect size, using Ward's minimum variance method. If SNPs were located within a 20 kb half-window of each other, only the SNP with the highest absolute cumulative effect size was included. The key shows the frequency distribution of SNPs across effect sizes.

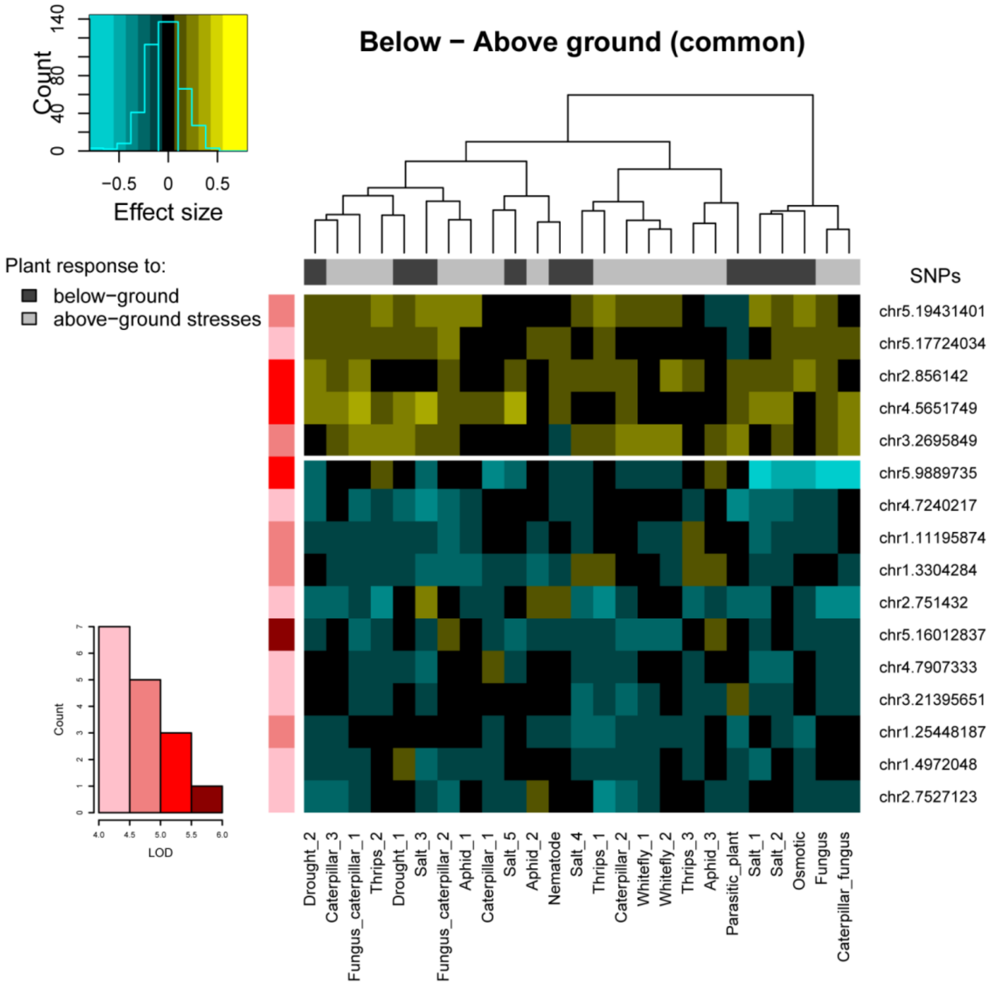

Fig. S10 Genetic associations common for plant responses to below- and aboveground stresses. Genetic associations were estimated with a contrast analysis using MTMM. Significant SNPs ($P \leq 10^{-4}$) for the common response are clustered according to trait-specific effects estimated from the full MTMM. If there was another SNP in LD that had a higher effect size, this SNP was used as representative for the LD block. Negative effect sizes (blue) were cases where the rare allele was associated with a detrimental effect on the plants, positive effect sizes (yellow) were cases where the rare allele was associated with increased resistance to the stress. The rare alleles of the top 5 SNPs are associated with enhanced resistance to above- and belowground stresses; the bottom 11 SNPs are associated with reduced resistance to above- and belowground stresses. Stresses are clustered according to SNP effect size, using Ward's minimum variance method. If SNPs were located within a 20 kb half-window of each other, only the SNP with the highest absolute cumulative effect size was included. The key shows the frequency distribution of SNPs across effect sizes.

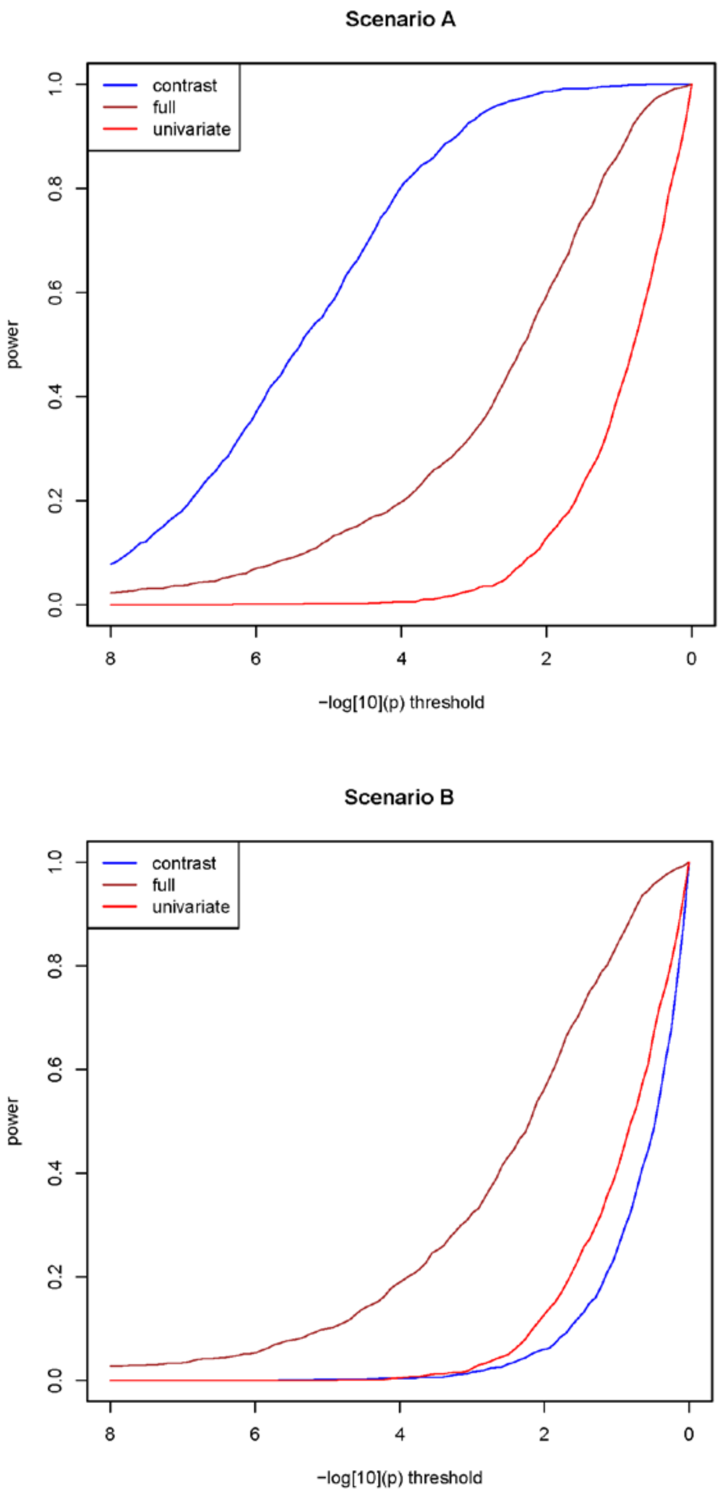

Fig. S11 Power of MTMM in simulations. Power of the full MTMM (brown), contrast MTMM (blue) and univariate analysis (red) as a function of P-value thresholds, in case of contrasting SNP-effects (Scenario A) and SNP-effects with random sign (Scenario B). Power was estimated based on 1000 simulations, which were performed as described in Methods S12.

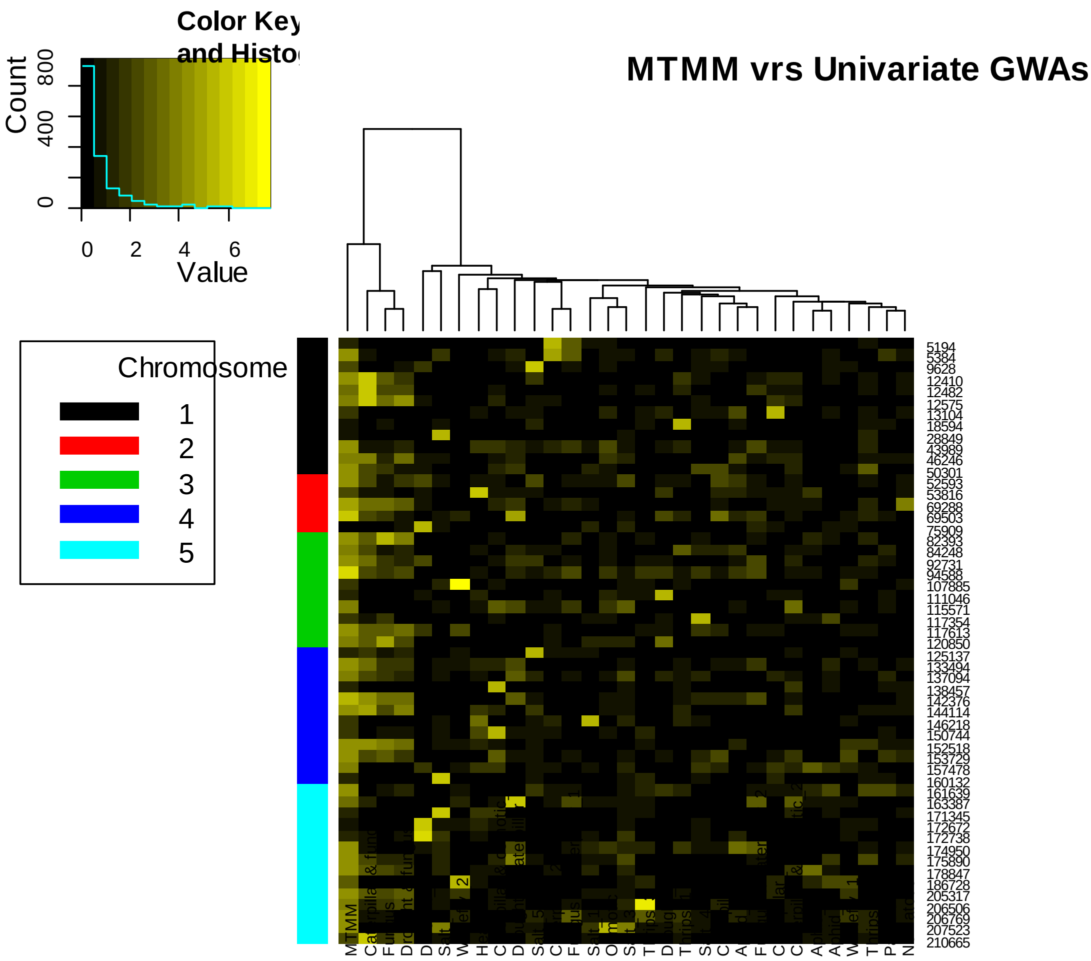

Fig. S12 Comparison of SNPs identified by MTMM and Univariate GWAS. Heatmap displaying *P*-values at -$\log_{10}$(P) scale of SNPs that were among the most significant in either MTMM (first column) or at least one of the univariate GWAS (subsequent columns). For MTMM a significance threshold $P \leq 0.0001$ was used. For univariate GWAS a significance threshold of $P \leq 4 \times 10^{-6}$ was used, following a multiple testing Bonferroni correction. Chromosomes are displayed with different colours in the left panel of the figure. Traits were clustered using hierarchical clustering (Ward). Per LD block only the marker with the highest effect size is shown. Markers within 20 kb half window size of each other were considered to be in LD.

References

Armengaud, P., Zambaux, K., Hills, A., Sulpice, R., Pattison, R.J., Blatt, M.R., and Amtmann, A. (2009). EZ-Rhizo: integrated software for the fast and accurate measurement of root system architecture. Plant J. **57,** 945-956.

Bac-Molenaar, J.A., Granier, C., Keurentjes, J.J.B., and Vreugdenhil, D. (2015). Genome wide association mapping of time-dependent growth responses to moderate drought stress in *Arabidopsis*. Plant Cell Environ. **39,** 88-102.

Cartieaux, F., Contesto, C., Gallou, A., Desbrosses, G., Kopka, J., Taconnat, L., Renoum, J., and Touraine, B. (2008). Simultaneous interaction of *Arabidopsis thaliana* with *Bradyrhizobium* Sp. strain ORS278 and *Pseudomonas syringae* pv. tomato DC3000 leads to complex transcriptome changes. Mol. Plant-Microbe Interact. **21,** 244-259.

Christ, B., Schelbert, S., Aubry, S., Sussenbacher, I., Muller, T., Krautler, B., and Hortensteiner, S. (2012). MES16, a member of the methyl esterase protein family, specifically demethylates fluorescent chlorophyll catabolites during chlorophyll breakdown in Arabidopsis Plant Physiol. **158,** 628-641.

Cline MS, Smoot M, Cerami E, Kuchinsky A, Landys N, Workman C, Christmas R, Avila-Campilo I, Creech M, Gross B, *et al*. 2007. Integration of biological networks and gene expression data using Cytoscape. Nature Protocols **2**: 2366-2382.

Fankhauser, C., Yeh, K.-C., Clark , J., Lagarias, Zhang, H., Elich, T.D., and Chory, J. (1999). PKS1, a Substrate Phosphorylated by Phytochrome That Modulates Light Signaling in Arabidopsis. Science **284,** 1539-1541.

Granier, C., Aguirrezabal, L., Chenu, K., Cookson, S.J., Dauzat, M., Hamard, P., Thioux, J.J., Rolland, G., Bouchier-Combaud, S., Lebaudy, A., Muller, B., Simonneau, T., and Tardieu, F. (2006). PHENOPSIS, an automated platform for reproducible phenotyping of plant responses to soil water deficit in *Arabidopsis thaliana* permitted the identification of an accession with low sensitivity to soil water deficit. New Phytologist **169,** 623-635.

Gravot, A., Deleu, C., Wagner, G., Lariagon, C., Lugan, R., Todd, C., Wendehenne, D., Delourme, R., Bouchereau, A., and Manzanares-Dauleux, M.J. (2012). Arginase Induction Represses Gall Development During Clubroot Infection in Arabidopsis. Plant and Cell Physiology **53,** 901-911.

Huang, M.S., Abel, C., Sohrabi, R., Petri, J., Haupt, I., Cosimano, J., Gershenzon, J., and Tholl, D. (2010). Variation of herbivore-induced volatile terpenes among Arabidopsis ecotypes depends on allelic differences and subcellular targeting of two terpene synthases, TPS02 and TPS03. Plant Physiol. **153,** 1293-1310.

Jubault, M., Hamon, C., Gravot, A., Lariagon, C., Delourme, R., Bouchereau, A., and Manzanares-Dauleux, M.J. (2008). Differential Regulation of Root Arginine Catabolism and Polyamine Metabolism in Clubroot-Susceptible and Partially Resistant Arabidopsis Genotypes. Plant Physiol. **146,** 2008-2019.

Kang HM, Sul JH, Service SK, Zaitlen NA, Kong SY, Freimer NB, Sabatti C, Eskin E. 2010. Variance component model to account for sample structure in genome-wide association studies. *Nature Genetics* **42**: 348-354.

Kloth, K.J., ten Broeke, C.J.M., Thoen, M.P.M., den Brink, M.H.V., Wiegers, G.L., Krips, O.E., Noldus, L., Dicke, M., and Jongsma, M.A. (2015). High-throughput phenotyping of plant resistance to aphids by automated video tracking. Plant Methods **11**: 4.

Lippert C, Listgarten J, Liu Y, Kadie CM, Davidson RI, Heckerman D. 2011. FaST linear mixed models for genome-wide association studies. *Nature Methods* **8**: 833-835.

Lu, X., Tintor, N., Mentzel, T., Kombrink, E., Boller, T., Robatzek, S., Schulze-Lefert, P., and Saijo, Y. (2009). Uncoupling of sustained MAMP receptor signaling from early outputs in an Arabidopsis endoplasmic reticulum glucosidase II allele. Proceedings of the National Academy of Sciences of the United States of America **106,** 22522-22527.

Luhua, S., Ciftci-Yilmaz, S., Harper, J., Cushman, J., and Mittler, R. (2008). Enhanced Tolerance to Oxidative Stress in Transgenic Arabidopsis Plants Expressing Proteins of Unknown Function. Plant Physiol. **148,** 280-292.

Maere S, Heymans K, Kuiper M. 2005. BiNGO: a Cytoscape plugin to assess overrepresentation of Gene Ontology categories in Biological Networks. Bioinformatics **21**: 3448-3449.

Matsumoto, T.K., Pardo, J.M., Takeda, S., Bressan, R.A., and Hasegawa, P.M. (2001). Tobacco and Arabidiopsis SLT1 mediate salt tolerance of yeast. Plant Molecular Biology **45,** 489-500.

Min, L., Li, Y., Hu, Q., Zhu, L., Gao, W., Wu, Y., Ding, Y., Liu, S., Yang, X., and Zhang, X. (2014). Sugar and Auxin Signaling Pathways Respond to High-Temperature Stress during Anther Development as Revealed by Transcript Profiling Analysis in Cotton. Plant Physiol. **164,** 1293-1308.

Mitchell, A., Chang, H.-Y., Daugherty, L., Fraser, M., Hunter, S., Lopez, R., McAnulla, C., McMenamin, C., Nuka, G., Pesseat, S., Sangrador-Vegas, A., Scheremetjew, M., Rato, C., Yong, S.-Y., Bateman, A., Punta, M., Attwood, T.K., Sigrist, C.J.A., Redaschi, N., Rivoire, C., Xenarios, I., Kahn, D., Guyot, D., Bork, P., Letunic, I., Gough, J., Oates, M., Haft, D., Huang, H., Natale, D.A., Wu, C.H., Orengo, C., Sillitoe, I., Mi, H., Thomas, P.D., and Finn, R.D. (2015). The InterPro protein families database: the classification resource after 15 years. Nucleic Acids Research **43,** D213-D221.

Molas, M.L., and Kiss, J.Z. (2008). PKS1 plays a role in red-light-based positive phototropism in roots. Plant, Cell & Environment **31,** 842-849.

Nalam, V.J., Keereetaweep, J., and Shah, J. (2012a). The green peach aphid, *Myzus persicae*, acquires a LIPOXYGENASE5-derived oxylipin from Arabidopsis thaliana, which promotes colonization of the host plant. Plant Signalling & Behavior **8:** e22735.

Nalam, V.J., Keeretaweep, J., Sarowar, S., and Shah, J. (2012b). Root-derived oxylipins promote green peach aphid performance on arabidopsis foliage. Plant Cell **24,** 1643-1653.

Nurmberg, P.L., Knox, K.A., Yun, B.-W., Morris, P.C., Shafiei, R., Hudson, A., and Loake, G.J. (2007). The developmental selector AS1 is an evolutionarily conserved regulator of the plant immune response. Proc. Natl. Acad. Sci. USA **104,** 18795-18800.

Obayashi T, Okamura Y, Ito S, Tadaka S, Aoki Y, Shirota M, Kinoshita K. 2014. ATTED-II in 2014: Evaluation of gene coexpression in agriculturally important plants. Plant and Cell Physiology **55,** e6.

Payne, R.W. (2009). GenStat. WIREs Comp Stat **1,** 255-258.

Rasmussen, S., Barah, P., Suarez-Rodriguez, M.C., Bressendorff, S., Friis, P., Costantino, P., Bones, A.M., Nielsen, H.B., and Mundy, J. (2013). Transcriptome responses to combinations of stresses in *Arabidopsis*. Plant Physiology **161,** 1783-1794.

Schneider, C.A., Rasband, W.S., and Eliceiri, K.W. (2012). NIH Image to ImageJ: 25 years of image analysis. Nature Methods **9,** 671-675.

Stracke, R., Werber, M., and Weisshaar, B. (2001). The R2R3-MYB gene family in *Arabidopsis thaliana*. Current Opinion in Plant Biology **4,** 447-456.

Stracke, R., Ishihara, H., Huep, G., Mehrtens, F., Niehaus, K., Weisshaar, B., and Barsch, A. (2007). Differential regulation of closely related R2R3-MYB transcription factors controls flavonol accumulation in different parts of the *Arabidopsis thaliana* seedling. Plant Journal **50,** 660-677.

Yanhui, C., Xiaoyuan, Y., Kun, H., Meihua, L., Jigang, L., Zhaofeng, G., Zhiqiang, L., Yunfei, Z., Xiaoxiao, W., Xiaoming, Q., Yunping, S., Li, Z., Xiaohui, D., Jingchu, L., Xing-Wang, D., Zhangliang, C., Hongya, G., and Li-Jia, Q. (2006). The MYB Transcription Factor Superfamily of Arabidopsis: Expression Analysis and Phylogenetic Comparison with the Rice MYB Family. Plant Molecular Biology **60,** 107-124.

Zhao, X., Wang, Y.L., Qiao, X.R., Wang, J., Wang, L.D., Xu, C.S., and Zhang, X. (2013). Phototropins Function in High-Intensity-Blue-Light-Induced Hypocotyl Phototropism in Arabidopsis by Altering Cytosolic Calcium Plant Physiology **162,** 1539-1551.
